# Emojis in Marketing and Advertising: A Systematic Literature Review

**DOI:** 10.3390/bs15111490

**Published:** 2025-10-31

**Authors:** Chrysopigi Vardikou, Agisilaos Konidaris, Erato Koustoumpardi, Androniki Kavoura

**Affiliations:** 1 Department of Digital Media & Communication, Ionian University, Leof. Antoni Tritsi 1, 28100 Argostoli, Greece; konidaris@ionio.gr; 2 Department of Cultural Heritage Management and New Technologies, University of Patras, University Campus, 26504 Rion, Greece; ekoustoumpardi@upatras.gr; 3 Department of Business Administration, University of West Attica, 12244 Egaleo, Greece; nkavoura@uniwa.gr

**Keywords:** emojis, digital marketing, communication, online consumer behaviour, social media, advertising

## Abstract

Studies examining emoji applications in digital marketing and advertising are characterized by considerable heterogeneity in their theoretical orientation, methodologies, and contextual factors. A domain-based systematic literature review with the Theory-Context-Characteristics-Methodology (T-C-C-M) framework following PRISMA guidelines was conducted to answer how emojis are researched in marketing, and a bibliometric review was constructed to shed light on important aspects. We found a field growing in volume yet immature, with a diversity of theories and methodologies used to explore the multiple roles of emojis. An analysis of explicit and implicit theories identified that almost a quarter of studies are atheoretical, and the mostly used theories are the Emotions as Social Information Theory (EASI) and the emotional contagion theory. Emojis are mainly researched in social media and in the travel and food industry. The most common methodological categories are experimental designs, with emojis used as independent variables in simple designs. Despite the focus on short-term outcomes (engagement, purchase intention), little attention was given to advertising and to field experiments, constraining ecological validity. Our study reveals the need for a robust theoretical framework that can explain the multiple functions of emojis, and EASI emerged as the leading theory to be tested more extensively.

## 1. Introduction

Emojis are small graphical symbols that serve as a visual language in online communication. They have been designed to enhance text communication in online environments, but, as human connection and communication progressively shifts from physical to digital spaces, emojis have gained prevalence because they express emotions and carry complex meanings in a simple way (Erle et al., 2022). Since 1988, when the first emoji-like set was released (Emojitimeline, n.d.), the evolution of emojis and their prevalence in online communication (and beyond) are undeniable. Approximately 4000 emojis have been approved by Unicode, the international standard for text encoding (Emojipedia, 2025a). While data about emoji usage across all platforms is not available, more than 166 million emojis have been copied from Emojipedia, the encyclopedia of emojis, and GetEmoji.com across all platforms, with 20 emojis copied each second (Emojitracker, n.d.). The prevalence is such that users have been asking platforms to include new emojis. In addition, platforms and software are publishing press releases about emoji inclusion in their updates, and artificial intelligence emoji generators are created to cover excessive demand (Feng et al., 2020; Nerdbot, 2025). Admittedly, as a paralanguage, emojis have been used in different ways by different generations, with Generation Z, people born from 1997 to 2012, excessively using emojis at work or to create secret codes and meanings with emojis (Emojipedia, 2025b; India Today, 2025; Abbasi et al., 2025; Zahra & Ahmed, 2025; Zhukova & Brehm, 2024).

Emojis are now embraced by marketers and advertisers in brand-to-consumer communication because of their unparalleled use to enhance it by conveying emotions and making it more vivid by describing objects, concepts, and situations with a simple icon.

In consumer behavior research, it is shown that people relate to brands as if they were people, and, at the same time, they expect brands to also satisfy emotional needs (K.-J. Chen & Lin, 2021; Delgado-Ballester et al., 2020). In this sense, emojis are also used in marketing strategy as tools to make the brand appear more human-like, what is called “anthropomorphism” (Agrawal et al., 2020).

Beyond emotional expression, other significant benefits are that emojis seem to assist in grabbing the attention of users in an era where the attention span is diminishing (Mark, 2023; Bai et al., 2019; Li et al., 2024; Stoianova et al., 2024) and seem to improve readability, the comprehension of messages, and decrease their ambiguity (J. Chen, 2023; Daniel & Camp, 2020; Boutet et al., 2021) because they serve as visual cues that make communication richer and easier to process when they are semantically and emotionally congruent with the text (Barach et al., 2021; Beyersmann et al., 2023).

Despite their increasing integration into digital marketing strategy and the fact that they have been studied extensively in linguistics and psychology (Setyawan & Musthafa, 2024; Bai et al., 2019), research on emojis in marketing and advertising is scattered across studies, and multiple contexts, diverse methods and theoretical frameworks are observed. **This fragmentation limits our ability to fully understand how, why, and with what results emojis are used in brand communication** and creates obstacles for researchers and marketers. Because of the methodological and theoretical diversity of the field, without a systematic approach, researchers are prevented from establishing and using standard methods to assess emoji effects. Moreover, a systematic review would mitigate the scattered findings in the field and would shed light on the potentially more productive theoretical lenses to be used. On the other hand, despite the large volume of studies, practitioners now cannot make informed decisions about the use of emojis in their marketing strategies unless they study all available studies. Furthermore, since lower emphasis has been given to advertising contexts, marketers do not have evidence on how emojis work in persuasive speech. To sum up, the field needs a systematic study that unites existing knowledge so that both academics and practitioners can have a solid base for their future work.

To date and to our knowledge, no systematic literature review has synthesized the findings for brand communication in marketing and advertising.

Following Palmatier et al. (2018), this is a domain-based systematic review that maps the landscape of emoji research in marketing. We conduct a bibliometric and systematic literature review to provide a consolidated understanding of the field, following the TCCM (Theory-Context-Characteristics-Methodology) framework with the PRISMA-SLR process to maximize clarity and transparency. This study’s main contribution lies in the mapping, organization, and categorization of the existing literature that helped us draw an overview of the current state of knowledge. The synthesis of key findings and the identification of critical research gaps in the theoretical development, methodological approaches, contextual coverage, and emoji characteristics examined are two other major contributions. Taking all of the above into account, we create a future research agenda that would advance the work of academics and practitioners.

## 2. Materials and Methods

### 2.1. Systematic Literature Review Protocol

This systematic literature review was conducted following the guidelines of the Preferred Reporting Items for Systematic Reviews and Meta-Analyses (PRISMA) statement (Page et al., 2021) and adhered to the principles of the Theory-Context-Characteristics-Methodology (TCCM) framework (Palmatier et al., 2018). The review protocol was registered on 30 May 2025 on the International Platform of Registered Systematic Review and Meta-Analysis Protocols (Vardikou et al., 2025b) under registration number INPLASY202405850 and is publicly available. Upon registration of the protocol, we ensured that no other similar research was reported in INPLASY.

### 2.2. Search Strategy

We developed a search strategy to identify relevant studies examining the use of emojis broadly in digital marketing and with a specific interest in advertising or e-commerce settings. The search was conducted in Scopus in May 2025, covering publications from 2002 to 2025. The choice of Scopus was justified to ensure that we had access to a larger pool of data and because it is a major database of publications’ metadata (Pranckutė, 2021; Khan et al., 2024). While Google Scholar has a larger pool of publications, Martín-Martín et al. (2018) have found that it also has a significant number of articles that are not published in journals.

First, we conducted a search of article titles, abstracts, and keywords to uncover if there was any other systematic literature review on emojis in marketing that we were not aware of, and the search returned none. Then search terms were combined using Boolean operators to capture all of the relevant literature. The Boolean search string used for titles, abstracts, and keywords was the following:

emoji OR emoticon OR emojis OR emoticons AND

“advertising” OR “ads” OR “advertisements” OR “marketing” OR “digital marketing” OR “ecommerce” OR “e-commerce” OR “e commerce”.

A total of 246 documents were initially found. When we applied the first criterion, English language, the search returned 233 documents, and when we filtered for articles published only in journals, the search returned 140 articles. To further justify our choice to include only journal articles despite the growing nature of our topic, we turned to other systematic literature reviews in marketing and consumer behavior that excluded articles published in proceedings or books, because the peer-review process in journals is rigorous (Alalwan et al., 2017; Khan et al., 2024). For the 140 remaining articles, we then performed the screening process, which is described in the next section.

### 2.3. Screening

All references retrieved were uploaded to Mendeley, a citation management tool, to manage duplicates. The screening of the deduplicated articles was performed using Rayyan, a web-based systematic review management platform that facilitates collaborative screening processes (Z. Yu et al., 2018; Ouzzani et al., 2016). Rayyan was selected for its several key advantages, including the following: (1) blind reviewing functionality that prevents reviewer bias during the screening process, (2) real-time collaboration features, (3) conflict resolution tools that highlight disagreements between reviewers, (4) tracking of screening decisions and rationales, (5) tagging of articles, and (6) desktop and mobile app availability to enable reviewers to collaborate seamlessly any time.

The screening was conducted by two independent reviewers who evaluated all identified articles against the predetermined inclusion and exclusion criteria. The eligibility criteria comprised specific inclusion and exclusion criteria to ensure the highest academic level of information. Inclusion criteria included the following: (1) The article is published in English; (2) the article is a peer-reviewed journal article; (3) the study is framed in a digital marketing context; and (4) the use of emojis is between the brand and consumer. Exclusion criteria were as follows: (1) The focus of research was on user-generated content; (2) the study has a purely linguistic analysis without a marketing or consumer focus.

Initially, titles and abstracts were screened for relevance. When the topic was not clear from the article’s title and abstract, a full-text screening followed. The two reviewers screened independently and, throughout this process, Rayyan’s blind review feature ensured that neither reviewer was influenced by the other’s decisions. When disagreements arose between the two primary reviewers, a third independent reviewer was consulted. All screening decisions, conflicts, and resolutions were systematically documented within the Rayyan platform to maintain transparency and reproducibility of the review process. Inter-rater agreement was “almost perfect” for title and abstract screening (κ = 0.851, 95% CI: 0.78–0.92), with reviewers agreeing on 130 of 140 articles (92.9% agreement). The 10 disagreements were resolved through discussion.

Regarding articles for which only the abstract was available, we contacted the authors to provide us with the full text and included them in the analysis. For the remaining articles with abstract-only, we included them in the descriptive bibliometric analysis but not in the TCCM analysis, as the required data about theories, methodologies, and characteristics were not sufficient.

**Scheme 1:**
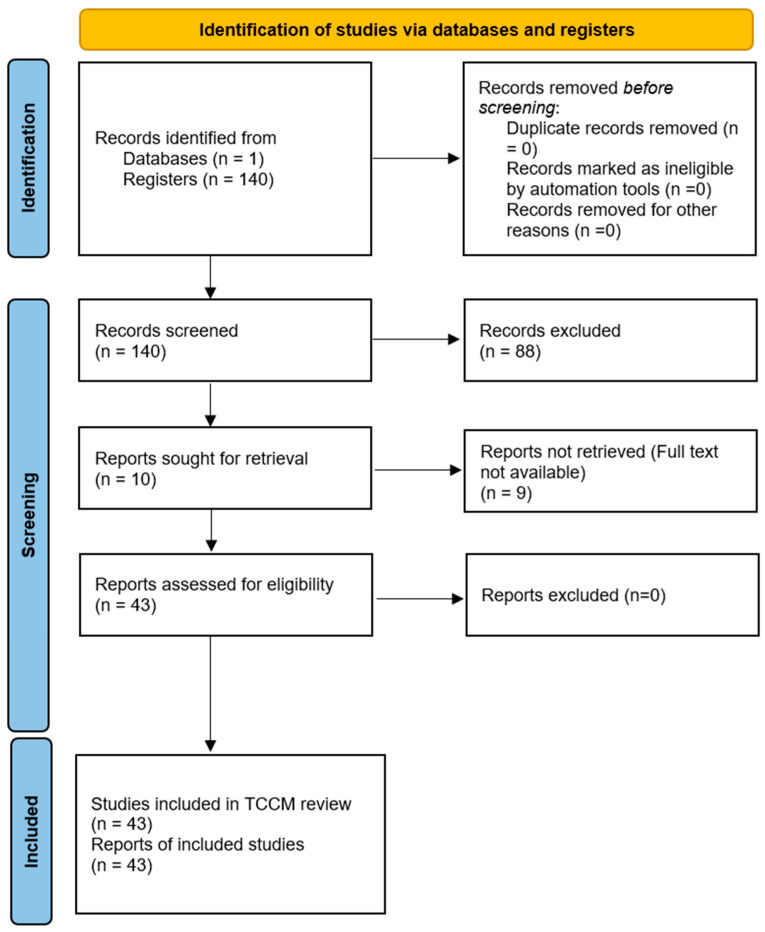
PRISMA flowchart, adopted from Page et al. (2021).

The entire selection process was carefully tracked and presented in the PRISMA-ScR flow diagram presented in Scheme 1 (Page et al., 2021) and indicated the number of records identified, screened, included, and excluded at each stage. The full list of the selected articles that were used in the T-C-C-M analysis is presented in Appendix A, Table A1.

### 2.4. Coding and Review Process

During the full-text review phase, eligible articles were tracked and organized using a standardized form in Google Sheets, which included listing details (number of listing, Rayaan number assigned), publication details (title, year, authors, and country of author affiliations), and the features necessary for the TCCM framework described below. TCCM permits research teams to “establish connections between various complex relationships” (Yeasmin, 2024, p. 3). We chose this particular framework because, as it has been shown, it makes reviews more impactful, and its effectiveness is demonstrated across marketing and consumer behavior domains (Paul & Rialp Criado, 2020).

The coding process for the “Theory” component was systematically implemented through a two-step approach to ensure comprehensive analysis of both explicit and implicit theories involved and implied in the studies. This choice was grounded in recent research (Schreiber & Cramer, 2024; Newman & Gough, 2019) that highlighted the hidden theoretical assumptions that are not explicitly stated but exist in some studies, which need to be documented and interpreted. However, this choice comes with limitations that will be discussed in the respective section.

For the first step, we coded all explicitly mentioned theories that were directly referenced, named, or formally cited by the authors within the included studies. The second step involved a more interpretive analysis to identify implicit theoretical concepts and underlying theoretical orientations that were not explicitly named. This phase was initially subdivided into two components: (1) primary theoretical focus identification—we tried to identify the broader domain or orientation (e.g., psychological, social, commercial, etc.); (2) implicit theoretical concepts extraction—we documented the theoretical concepts that emerged from the studies’ research questions, frameworks, literature reviews, and variable relationships. This theoretical coding process helped us ensure that we recorded the formally used theories together with the implied theoretical foundations.

Responding to the reviewer’s comment, we took additional action for triangulation of the results with other studies to minimize bias. More specifically, first, we triangulated with articles published by the same authors to understand if, in other papers, they explicitly use a relevant theory. Second, we triangulated with other articles that cite the initial studies. In this step, we found all the articles of our study in Google Scholar, and then we studied all the cited articles that were published in English and were relevant to the initial study by including filters for words like “emoji” or “emoticon”.

As this step involved manual coding and interpretation, we incorporated the principles of Thompson et al. (1989) to minimize bias and make our subjectivity as transparent as possible. In this direction, we used a Gioia codebook (presented in Appendix A, Table A2) where we systematically put the second-order implicit concepts together with the first-order text parts from which we implied them, and then we tried to aggregate them to a theoretical concept or theory. Each article could have multiple codes. Interpretations were triangulated with the explicit theories extracted from the main body of articles.

If an excerpt was ambiguous for coders and we could not assign an implicit concept, we did not use it in the final analysis. If we were not able to extract any implicit concepts, we conservatively categorized the articles as “Atheoretical”. This happened in one out of the ten articles, included in Appendix A, Table A2.

However, even with these measures, we expect that bias inherently exists; thus, the results presented in the respective Section 4.1.2 are subjective and may be treated as only one reading out of the multiple possible readings of the theoretical landscape in emoji research.

For the “Methodology” component of TCCM, we recorded the broader category of each research study and a sub-category that was developed with relevance to the primary category. For mixed methods studies, beyond the sub-categories, we also separately coded them if they included a field or lab experiment. In addition, for the experimental studies category only, we kept track of sample characteristics, such as gender, age/generation, and sample size.

For the “Context” component of TCCM, we recorded information about the marketing channel, platform, industry, and country of experiment/analysis. For the “Characteristics” component, and where applicable, we introduced the following fields to uncover emoji dimensions that are potentially important: (1) emoji type (face, object, gestures, symbols, and animals); (2) emoji function (emotional expression, emphasis, word replacement, and decoration); (3) emoji sentiment; (4) emoji placement; (5) emoji frequency; (6) emoji combinations; (7) independent variables; (8) dependent variables; (9) moderating variables; and (10) mediating variables.

In sum, we employed a carefully designed and executed screening and coding process to create a robust foundation for our analysis and future directions. The meticulous methodological rigor employed in this review, with the combined use of the TCCM framework, Prisma reporting, Rayaan screening, and coding in Rayaan and Google Sheets, hopefully ensures academic integrity, clarity, and transparency and is reproducible by other researchers.

## 3. Results of Bibliometric Review

### 3.1. Overview

For our bibliometric analysis and the publication trends, we performed the analysis in Google Sheets (Google LLC; Mountain View, CA, USA) and VOSviewer–Version 1.6.20 (Centre for Science and Technology Studies, Leiden University, Leiden, The Netherlands), a bibliometric software package that shows the relationships of the publication data. Because the scope of the bibliometric review is broader and we are trying to map the full field and the relationships of data, we included all 140 articles that Scopus returned before the manual screening process.

#### 3.1.1. Publication Trends

**Figure 1:**
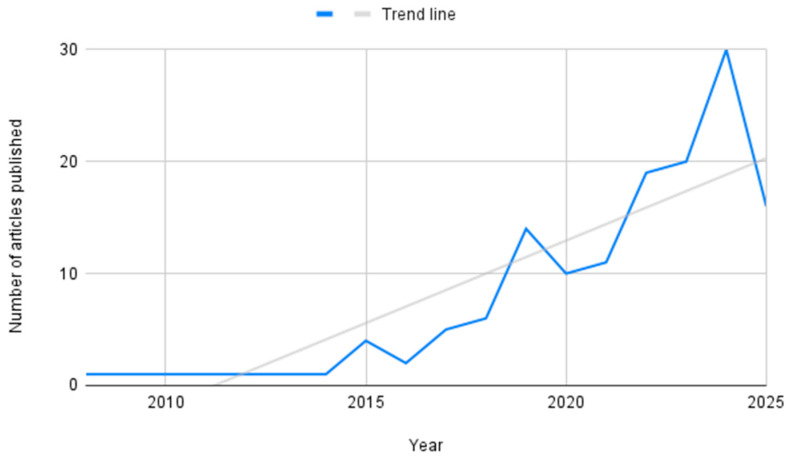
Annual publication trends; data from Scopus.

In Figure 1, the annual publication trends are presented. First, it is clear that there was no emoji research in marketing before 2008. There is a clear separation between the early stages (2009–2014) with one study per year, a growth phase from 2015 to 2018, and an acceleration phase from 2019-today. The year 2024 was a peak year, and 2025 shows a decline because of incomplete data. In sum, emoji research in marketing and advertising is steadily growing, as the trend line shows.

#### 3.1.2. Article Classification

Table 1 shows the 10 most cited articles with their authors, year of publication, and number of citations. The average number of citations of the 10 articles is 139 citations, while the average of all 140 articles is 20 citations, with a ratio of 7 to 1. These ten articles together account for 49.9% of the total citations, showing that they serve as the foundation in a field that is quickly evolving. Obviously, in the list we do not have articles published after 2020 despite their larger volume, which clearly shows a citation delay, probably because of long submission-to-publication periods.

#### 3.1.3. Journal-Wise Classification

In Table 2 we present the top 17 journals according to their number of articles in emoji research in the context of marketing and advertising. Approximately 44 articles are distributed between the top 17 sources, and the rest are dispersed among multiple sources. We notice that journals with a business, technology, and psychology focus appear in the list, depicting a landscape where emojis in marketing are viewed within diverse disciplines.

#### 3.1.4. Conceptual Structure

**Figure 2:**
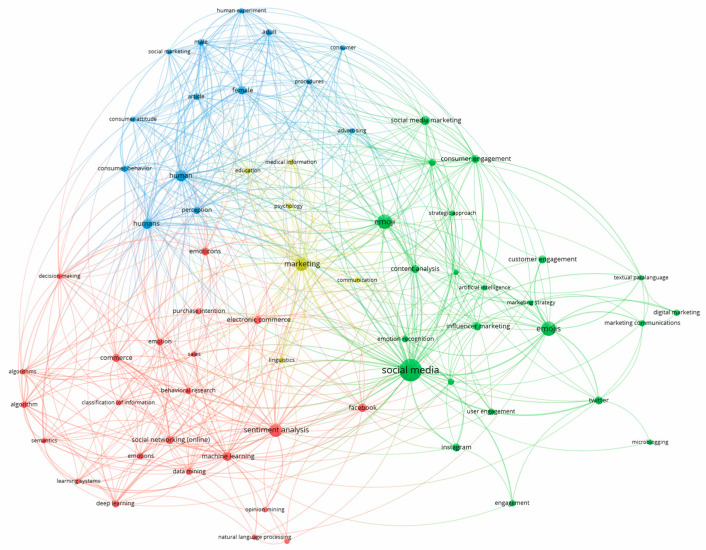
Keyword (all) co-occurrence with full counting. Keywords with more than 3 occurrences are included. Image created with VOSviewer. Source: Authors.

**Figure 3:**
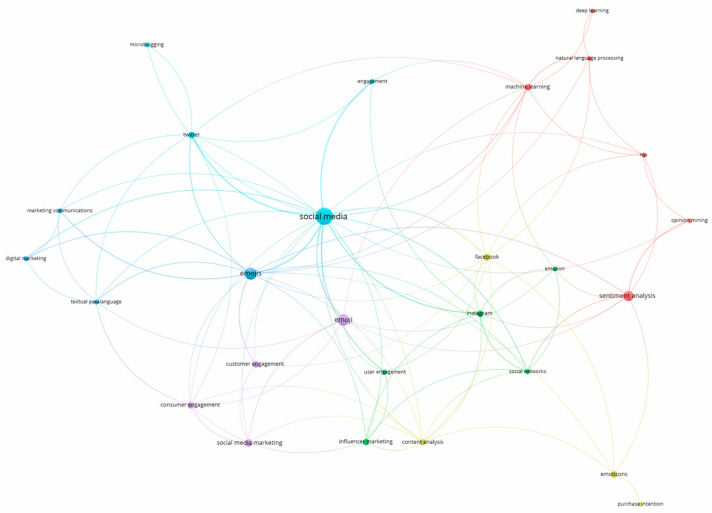
Author keyword co-occurrence with full counting. Keywords with more than 3 occurrences are included. Image created with VOSviewer. Source: Authors.

In Figure 2 and Figure 3, we see the keyword co-occurrence analysis of the field for all keywords (Figure 2) and author keywords (Figure 3). First, once more, we observe the **interdisciplinary nature of the field**. In Figure 2, the green cluster represents the business perspective (social media, digital marketing, and marketing communications), which seems to have a slightly central position, showing that the primary point of view in emoji research in marketing is commercial and with a focus on social media, the natural environment where emojis are used. The blue cluster has “human” at its core, representing the psychological aspects examined (e.g., perception, demographic variables), while the red group is more technology-focused, concentrated around terms such as “sentiment analysis”, “machine learning”, “data mining”, and “algorithms”.

In the same cluster, we see an interest in operationalizing emotional understanding with emojis in a social media context. Its proximity to the blue cluster shows that there is an effort to bridge the gap between traditional psychological research with technological advancements or that there are efforts in the classification of emojis based on their emotional aspects.

The yellow cluster in the middle of the graph summarizes the interdisciplinary nature of the field. Emojis are researched in marketing for their psychological and linguistic properties.

In Figure 3, we present author keywords, and we notice the authors’ practical approach in keyword selection. There is now a higher emphasis on commercial aspects, as authors use keywords about specific platforms and commercial concepts, probably to make their research discoverable by marketers.

**Figure 4:**
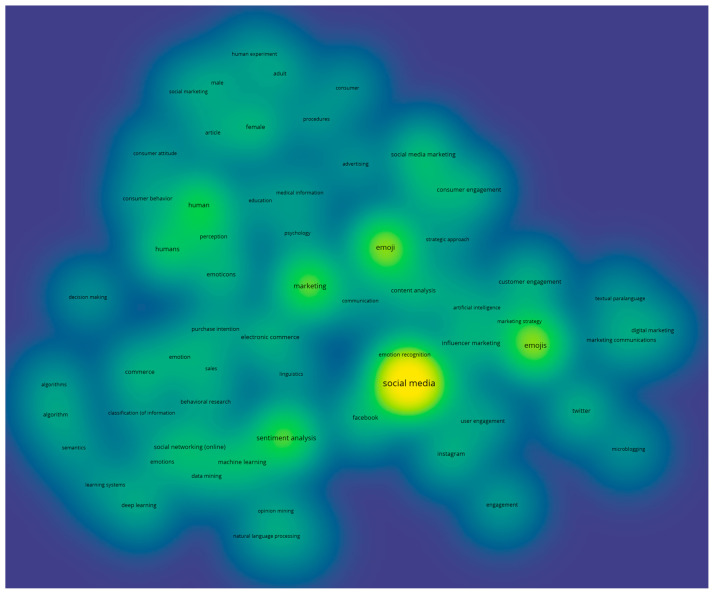
Keyword (all) co-occurrence—density map. Keywords with more than 3 occurrences are included. Image created with VOSviewer. Source: Authors.

In the density heatmap presented in Figure 4, differences between the keywords “sentiment analysis”, “emotions”, and “semantics”, in terms of the variety of keywords, their density, and distance, do not go unnoticed. **While emojis carry both emotion and meaning, it seems that there is a preference to study their emotional aspects** (keywords: emotion, “emotion recognition”) **rather than their semantic ones** (keywords: “semantics”, “opinion mining”), which further means that we have been focusing on an emotional rather than a cognitive point of view. Last, while we had a particular focus on advertising, we can notice its very low density and its distance from all other concepts, showing that it has received very little attention.

#### 3.1.5. Country Analysis

The top--cited countries are presented in Table 3. The top three countries (United States, China, and India) appear to have the most impactful work, accounting for 52.86% of all countries in the dataset. We also notice equally distributed citations between European (United Kingdom, Netherlands, and France) and Asian countries (South Korea, India, Singapore, etc.), with smaller countries appearing to have opportunities for high citations.

**Figure 5:**
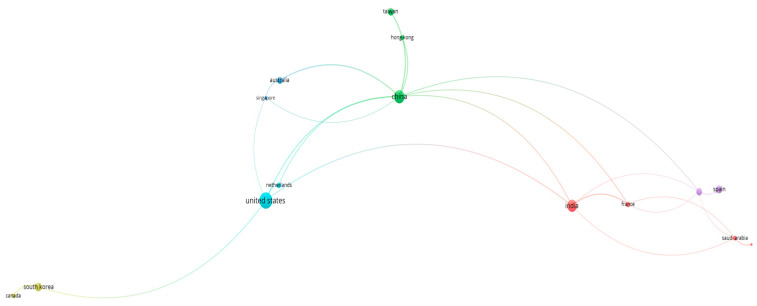
Countries’ co-authorships with fractional counting. Fifteen countries with minimum 1 document and at least 10 citations are included. Adjusted view with a focus only on the countries present in a network. Created with VOSviewer. Source: Authors.

Looking at the next graph of co-authorship (Figure 5), we obtain a more comprehensive picture of country dynamics in the field. From the bigger nodes, we notice that authors from these countries have established collaborations, resulting in three clear clusters. From the above findings of the overview, the **United States was the most prominent in terms of citations, but India and China have a central bridging role with collaborations**.

## 4. Results of T-C-C-M Analysis

### 4.1. Theoretical Perspectives (T)

#### 4.1.1. Explicit Theories

Across the 43 studies, the analysis of the explicitly mentioned theories gave 52 different theoretical references, presented in Table 4, indicating that some of the studies used multiple theoretical models and that researchers have been drawing results from diverse theories. A quarter of the studies (n = 10) were atheoretical, which is a pattern found before in consumer behavior systematic reviews (e.g., Bhardwaj & Kalro, 2024). There is prioritization of empirical findings over theoretical foundations, with 50% of the machine learning studies not using an explicit theory, while the more “traditional” content analysis studies prefer the standardized approach of grounding decisions in theoretical models. **The combination of atheoretical studies with a wide mix of theories used indicates that the field is still in its early stages of theoretical development** and that there is not a single framework explaining the multitude of emoji characteristics in brand communication.

Our analysis of the most prominent theories used in the studies for our sample is presented below in Table 4.

*(a)*  *Emotions as Social Information Theory (EASI)*

The EASI theory (Van Kleef, 2009) explains that the emotion conveyed and transferred by someone affects the behavior of the observer in dual pathways: inferential processes and affective reactions. The level to which the observer will be influenced by the two mechanisms depends on their information processing.

To our knowledge, there is only one study that has tested if the EASI theory is valid for emojis (Erle et al., 2022), and it was partially confirmed, since the results showed that, **while the affective pathway had the same results as in face-to-face communication, the inferential pathway probably works in different ways**. This second observation could be linked to the initial idea of Van Kleef (2009) that the inferential pathway works when the observant can accurately perceive the meaning.

One study (Maiberger et al., 2024) confirmed the inferential pathway and found that using the facial emoji as a replacement increased ambiguity and thus hurt the inferential process. Another study (Madadi et al., 2024) has found that emojis may make the meaning of the message more complex and hurt processing, and concluded that context moderates effectiveness.

*(b)*  *Media Richness Theory (MRT)*

Media Richness Theory was originally developed by Daft and Lengel (1986) and categorizes media based on their capacity to process information, with richer media being more effective for complex tasks. MRT is used in a corporate context to give insights about platform characteristics that enhance or constrain corporate communication effectiveness. Under this theory, emojis promote richer communication and facilitate better processing. In the studies examined, MRT was mainly used in content analysis studies.

*(c)*  *Hofstede’s cultural dimensions*

Hofstede’s cultural dimensions theory (Hofstede, 2011) was the only cross-cultural theoretical framework in our sample of studies. In our corpus, it was used in two studies employing content analysis but with contradictory results. Neel et al. (2023) found no interaction between nationality and emoji sentiment across three Western cultures (American, British, and Danish). P. Wang and McCarthy (2020), on the other hand, found cultural differences between Australians and Singaporeans for different message types. These contrasting findings imply that **different cultures perceive corporate messages in different ways, but sentiment is more “universal”.**

*(d)*  *Emotional contagion*

The emotional contagion theory (Hatfield et al., 1992) is based on the idea that people, from their infancy, “tend to mimic facial expressions… of those around them”, and through that automated copying of expressions, “they catch other people’s emotions” with “complex cognitive processes” (p. 154) but also with unconscious mechanisms. While it was applied for the positive effect of facial emojis (Das et al., 2019), it cannot explain why emojis bring emotions to users.

*(e)*  *Other studies*

The use of 29 theoretical frameworks indicates that **to understand the multiple traits of emojis, we need to rely on psychology, cognitive theories, and relationship marketing**. There is a notable absence of widely used theories in consumer behavior research, such as the Stimulus-Organism-Response model and the Theory of Planned Behavior. This choice is rationalized because of the focus on the immediate outcomes of emojis.

#### 4.1.2. Implicit Theories

Among the 11 atheoretical studies, triangulation (Appendix A, Table A2 and Table A3) has revealed that 5 studies were atheoretical and seven frameworks were used: Affective Science/Emotional Contagion (n = 2), Construal Level Theory (n = 1), Elaboration Likelihood Model (n = 1), Information Processing theories (n = 2, one linked to content complexity), and Anthropomorphism (n = 1, explaining effects via human face resemblance).

In the mix of explicit and implicit theories, we now see a more balanced approach to affective and cognitive frameworks, a finding that is in line with researchers’ choices about mediators, presented in 4.3. Results are discussed in Section 5.1.

### 4.2. Context (C)

#### 4.2.1. Geographic Distribution

The field is geographically spread across 21 countries, yet there are some dominant forces, namely the United States and China, reflecting their broader focus on consumer behavior research (Scopus, 2025). South Korea shows a strong focus on the field of emojis, probably because of its technology-driven research culture and a clear direction towards collaboration between the industry and institutions (Chung et al., 2022; Cho, 2014). The presence of five multi-national studies reflects the need for cross-cultural comparison (Appendix A, Table A4).

Despite preliminary findings about universal emoji usage in three Western countries (Neel et al., 2023), there is also contradictory evidence about significant differences across cultures and countries. For example, the study by P. Wang and McCarthy (2020) found that there are cultural differences in the perceived credibility of the source in Australian and Singaporean audiences.

#### 4.2.2. Industries

From an industry perspective (Appendix A, Table A5) there is also remarkable diversity. The prevalence of the travel industry could be owed to the general growing interest in tourism marketing in the last years (Scopus, 2025) or to the more emotional side of the industry, ideal for leveraging the emotional and anthropomorphic properties of emojis that enhance the message and influence consumer behavior (Hosany & Prayag, 2013; Ramos et al., 2013; Walters et al., 2012; Han et al., 2019; Christou et al., 2020; Ding et al., 2022). In this corpus of studies, emojis seem to have only favorable results in terms of user engagement, hotel selection, and eWom (Hsu & Chen, 2020; P. Wang & McCarthy, 2020; Orazi et al., 2023; X. Wang et al., 2023a; J. Yu et al., 2024). However, effectiveness may be lower for premium/luxury hotels. Research in this sector has uncovered the need to select emojis that are congruent with the topic and type of content.

In electronics, interestingly, we have limited attempts to replicate the work of Das et al. (2019) with the same product (digital camera), while many studies from our sample have used the work of Das et al. as the foundation for their work. Results about the effect of emojis on purchase intention are contradictory.

Surprisingly, **the fashion industry is underrepresented** despite the growing research interest in fashion marketing, especially in the past two years (Scopus, 2025). Given its hedonic nature and the role of emotion in it, we expect to see more studies about emojis in fashion marketing (Bishnoi & Singh, 2022; Chan et al., 2015; Zahari et al., 2022). A similar note must be made for the beauty industry, which is inherently emotional due to its hedonic nature (Hashem et al., 2020; Radhi et al., 2024; Diwoux et al., 2024; Courrèges et al., 2021).

**Traditional sectors (e.g., furniture, real estate, automotive, and services such as telecommunications or insurance) and emerging ones (e.g., fitness/wellness, entertainment) are not explored**, a finding probably owed to the small sample and the lower volume of research in these fields.

The significant portion of studies (seven cases) without specific industries suggests that emoji research transcends industry boundaries or focuses on general consumer behavior patterns.

#### 4.2.3. Marketing Channels and Platforms

More than half of the studies are located in a social media context, and there is a substantial body of literature without a specific channel. Research seems to focus more on general emoji principles than on channel-specific applications (Table 5).

There is also **notable underrepresentation of “Advertising”** (not including social media advertising). It could be owed to technological or resource barriers such as restrictions on platforms not allowing advertisers to use symbols (Google, n.d.), or financial resources and industry partnerships needed to run field experiments.

Email marketing is also underrepresented, a finding that is particularly puzzling because conducting field experiments would be easy and less expensive, as platforms like Mailchimp give marketers multivariate testing tools (Vardikou et al., 2025a).

The finding that most of the studies did not focus on a specific platform reinforces the idea that emoji research in marketing communication is still in its infancy, trying to build basic knowledge. As the field progresses, we may see more refined approaches with specific platform focuses.

In Table 6, we observe a focus on social media on the three platforms that represent the core of the social media ecosystem: Twitter/X (combined), Facebook, and Instagram. The first position of Twitter/X is surprising, given that it is not the biggest platform worldwide, and could be explained by accessibility to its academic-friendly API until 2023, which gave researchers free access to data (Coalition for Independent Technology Research, 2025).

On the other hand, Facebook, the leading platform worldwide in user base (Statista, 2025), has accessibility barriers, as it required researchers to apply to the Inter-University Consortium for Political and Social Research ICPSR (Meta, 2025b). Moreover, its dedicated platform Crowd Tangle was deactivated in 2024 (Meta, 2025a), making data acquisition even more complex. A limited representation of emerging platforms like TikTok (one study) suggests a delay in capturing the trends and in publishing.

### 4.3. Characteristics (C)

**Scheme 2:**
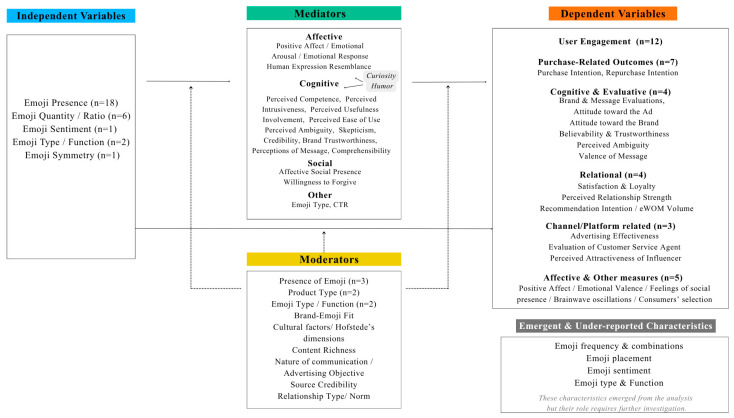
Characteristics: Independent, dependent, moderators, and mediators. Source: Authors.

A conceptual framework is developed to categorize the variables identified in the analysis. Alongside the traditional relationships (independent/dependent variables, moderators, and mediators), we present other emoji characteristics that emerged in our analysis and that could potentially serve as independent variables or moderators. The key variables are presented in Scheme 2.

We consider the findings in Section 4.3.8, the **“anthropomorphic bias”** towards the selection of facial emojis and the **“positivity bias”** towards the selection of happy emojis, to be two compelling findings in this section.

#### 4.3.1. Independent Variables

Our analysis of independent variables, presented in the Appendix A, Table A6, revealed diverse characteristics and a striking pattern. **Emoji presence/absence was the dominant independent variable,** and most studies agree that the presence of emojis brings the desired consumer responses and evaluations. Emoji presence has been linked with higher post engagement (Taba et al., 2023), affect transfer (Das et al., 2019; Smith & Rose, 2020; Neel et al., 2023), feelings of social presence, and positive evaluations (Park & Sundar, 2015). They have also been linked to customer selection when they are presented as subliminal cues for 1 millisecond (Hsu & Chen, 2020).

There is no consensus on whether the presence of emojis is linked with higher purchase intentions. Even for the same hedonic product and in the same context of sponsored ads, emoji presence can be linked with higher purchase intentions (Das et al., 2019) or lower purchase intentions when the ad is perceived as more personalized (Lee et al., 2021). It seems that the relationship is moderated by the valence of emotion (Das et al., 2019; Mladenović et al., 2022).

Given the limited focus on advertising and the methodological differences in the studies, the replication of such experiments for hedonic and utilitarian products would be beneficial to uncover the dynamic. However, when measuring valence, it seems that users’ self-reports of valence may differ from implicit biometric measures (Rúa-Hidalgo et al., 2021).

There is contradicting evidence that emojis may bring negative responses, for example, by hurting seriousness and authenticity (Madadi et al., 2024). Additional research would be necessary to understand if the self-reported measures match the real responses.

Next, we see a **clear quantitative focus** on independent variables such as the number of emojis, number of facial emojis, and the emoji ratio. A higher number of emojis has been linked with higher review trustworthiness (Huang et al., 2021), while on Twitter, there seems to be an optimal number from 0 to 3 emojis, as a higher number decreases engagement (Guzmán Ordóñez et al., 2024), and in other studies, there was no effect of the emoji ratio on engagement (Yang & Shin, 2021). So far, it seems that “more is not better”, and we need to study the optimal emoji ratio.

Some other studies explored the categorical aspects of emojis, indicating **interest in exploring how different categories of emojis function**. Non-facial emojis seem to increase eWom volume (Orazi et al., 2023), and asymmetrical facial emojis are linked to higher user engagement because the human expression resemblance is higher (Hewage et al., 2021). In the context of customer service recovery, emojis with a negative sentiment may lead to higher satisfaction and repurchasing behavior (Ma & Wang, 2021).

Also, we notice a small percentage of studies have an independent variable not related to emojis, probably showing initial efforts to understand antecedents of emoji usage or emoji reactions. More details will be presented in the respective sections of moderators and mediators.

The above findings reinforce what is also mentioned in other parts of our analysis, that the field remains in an exploratory phase where basic effects from emoji presence and emoji numbers are tested.

#### 4.3.2. Dependent Variables

We employed a two-stage approach for the analysis of dependent variables. We first documented all dependent variables (Appendix A, Table A7), and we created a consolidated table (Table 7) based on similarity and conceptual coherence so that the broad categories are clear, while maintaining granular information.

*User Engagement/Consumer Engagement*

The primary category of dependent variables is User Engagement/Consumer Engagement (34.29%), showing a focus of marketing researchers on the effects of emojis in user interactions with content. Based on the synthesized evidence, emoji usage produces mixed but generally positive effects on user engagement. **Posts with emojis receive more likes** Taba et al. (2023) and TikTok videos with emojis **are shared more** (Einsle et al., 2024).

However, findings indicate that **emoji effectiveness depends on strategic factors including emoji selection, content type, quantity, content placement, and source credibility**. The selection of the right emoji or emoji category for the content type is important, as the same emoji brings different results when placed in different topics (X. Wang et al., 2023a, 2023b; J. Yu et al., 2024). For example, engagement is higher when emotional emojis are used with aesthetic content and when semantic emojis are used in promotional content (X. Wang et al., 2023a)

In contrast, in some cases, emojis decrease engagement, for example, by **making diversity and inclusion posts less effective** (Bombaij & Mokarram-Dorri, 2024), by decreasing open rates in email marketing when they are artifact emojis (C. H. Kim et al., 2024), or by affecting **how users perceive message sources (Balaji et al., 2023)**, and they make firm-generated content less effective compared to employee-generated content. Generally, authors grounded this result in the loss of expertise or the increase in skepticism.

*Purchase-Related Outcomes*

The second category (20%, n = 7) of dependent variables is Purchase-Related Outcomes. This finding is aligned with results from keyword analysis (Section 3, Figure 2 and Figure 3). Given the underdevelopment of the field, it seems to be a theoretically premature emphasis.

The analysis showed that emojis mostly **bring higher purchase intention because, firstly, they increase trust and consumer engagement** (Duffett & Maraule, 2024) and **secondly, they increase positive affect**, but this result is valid for hedonic and not utilitarian products (Das et al., 2019; Mladenović et al., 2022). Interestingly, most of these studies focused on Generation Z, indicating a research gap.

We also have totally contradicting evidence for the same product (camera), where emojis bring lower purchase intentions (Lee et al., 2021), calling for the replication of these studies in field experiments. Another interesting study in a gift-giving setting (Huang et al., 2021) had mixed results on purchase intention, in that facial emojis had stronger effects on purchase intentions only in communal (versus exchange) relationships, meaning only when the gift giver does not expect something in return. All the above findings indicate that additional research is necessary on the factors affecting the relationship between emojis and purchase intention, as **the context of the purchase matters, and relationship dynamics could also play a role**.

*Specialized Measures*

Specialized measures (brainwave oscillations, construal level, difference between implicit and explicit measures of valence, feelings of social media presence, etc.) are dependent variables in 14.29% of the studies. This sophistication could also be explained by the presence of multiple studies exploring emojis in a psychological context without a marketing focus (Bai et al., 2019; Kaye et al., 2017; Riordan, 2017a).

As the work of Hsu and Chen (2020) suggests, emojis used repeatedly as subliminal cues for 1 millisecond can bring positive consumer responses (hotel selection). Linked to the above, this work paves the way for **the use of neuroimaging tools to understand hidden responses that precede engagement and purchase intention**.

*Other variables*

Consumer preferences and evaluations are mostly positively affected by the use of emojis, but results are fragmented. Review trustworthiness is positively affected by a higher number of emojis (Huang et al., 2021), consumers’ selection of hotels is positively affected by the subliminal presence of a face emoji (Hsu & Chen, 2020), and evaluation of communication with employees of the company and influencer attractiveness are also positively affected by the presence of emojis (Park & Sundar, 2015; Balaji et al., 2023; Guo & Wang, 2024).

In an interesting study (Boman et al., 2023), emojis with a dark skin tone have led to higher consumer preference of conservative brands through higher brand advocacy.

Emojis are also linked to higher customer satisfaction when used in post-purchase settings (Indwar & Mishra, 2022) and in customer service recovery (Ma & Wang, 2021).

In sum, the distribution of dependent variables in emoji research shows, firstly, pluralism and secondly, a **strong practical focus on short-term behavioral outcomes** (user engagement and purchase intentions). As knowledge progresses over time, we expect to see more studies with a long-term focus on the relationship impact of emojis rather than direct user engagement.

Findings have revealed that emojis bring generally positive effects on user engagement, purchase intentions, and consumers’ evaluations and preferences, but this effectiveness is highly dependent on the context. Contradictory findings for the same product imply methodological differences that have to be addressed in the future.

#### 4.3.3. Moderators

Nearly **half of the studies explored had no moderating variables** at all, and most of the studies focused on basic factors, such as the presence/absence of emojis, emoji combinations, product type (utilitarian/hedonic), and presence of a profile picture (Appendix A, Table A8).

The **presence of emojis has been found to act as a moderator between the content and its effectiveness, for example,** by moderating the relationship between the valence of content and the effectiveness of advertising through the reinforcement of positive thumbnail effects and of negative title effects (Li et al., 2022) or between the message source (employee-generated vs. firm-generated content) and social media engagement and trust (Balaji et al., 2023).

In cause-related marketing, emoticons attenuate the interaction between the donation size and construal level (Yoo et al., 2018), and they also reduced the perceived monetary sacrifice and increased positive responses. This finding creates opportunities for emoji usage in cause marketing. However, the above results are valid for emojis that complement the text, as emojis that replace words have been linked to low effectiveness (Maiberger et al., 2024).

As already discussed, context matters in emoji effectiveness. Regarding the product type, hedonic products present increased effectiveness with emojis (Mladenović et al., 2022; Das et al., 2019). The product type has been previously found to moderate consumer behavior variables’ relationships (see Ren & Nickerson, 2019; Kivetz & Zheng, 2017). Referring to Section 4.2 and excluding the food industry (three cases), which is both utilitarian and hedonic (Coimbra et al., 2023), we see a balanced distribution, aligning with recent neuroscientific data showing that, in both types, there are emotional responses from consumers (Bettiga et al., 2020).

Variables related to relationships and the brand have also served as moderators. For communal (versus exchange) relationships, the effect of emojis in the positive affect, customer satisfaction, and repurchasing behavior is higher (Smith & Rose, 2020; Ma & Wang, 2021). Emojis provide less benefits to premium sellers (Orazi et al., 2023), and there seems to be a brand–emoji fit, a campaign objective–emoji fit, and a content–emoji fit that moderate effectiveness (Rúa-Hidalgo et al., 2021; X. Wang et al., 2023a; C. H. Kim et al., 2024), indicating that the congruence of the emoji is important.

However, congruence should be further explored, as it may have even more types, such as semantic, emotional, and cultural. Interestingly, the only study exploring nationality as a moderator found no differences between three Western countries (Neel et al., 2023).

#### 4.3.4. Mediators

Results from the analysis of the mediating factors are presented in the Appendix A, Table A9. Again, more than half of the studies (57.1%) explored no mediating variables, which reveals a **critical methodological gap**.

In the rest of the studies employing a mediation relationship, we observe more affective and cognitive factors and less social and ideological factors. **Affective factors,** from the more generic positive affect to the more specific emotional arousal, show a primary recognition that emojis bring desired results through emotional pathways, consistent with the EASI theory and emotional contagion theory.

The human expression resemblance (anthropomorphic nature) of emojis, pleasure and arousal, and affective social presence are facilitating mediators that have all been linked to the higher effectiveness of emojis, but this finding has only been explored for facial emojis that carry emotion (Smith & Rose, 2020; Hewage et al., 2021; X. Wang et al., 2023b; Park & Sundar, 2015).

Suppressing **cognitive mediating factors** include processing fluency, comprehensibility, skepticism, perceived competence of the brand, and childishness and trustworthiness of the brand (C. H. Kim et al., 2024; Orazi et al., 2023). It seems that cognitive responses may be considered better fits as mediators in cases of semantic emojis, but this finding has to be further explored because it relies on a dichotomy between emotional and semantic aspects that may not exist (X. Wang et al., 2023a).

Perceived usefulness, perceived ease of use, and involvement with emojis may also serve as enabling cognitive mechanisms that explain the positive relationship of emojis with purchase intentions but given the limited sample with only South African Generation Z people, additional research is necessary (Duffett & Maraule, 2024). Perceived intrusiveness was not confirmed as a constraining mediator between emoji usage and purchase intentions (Lee et al., 2021).

Up to here, we realize that **the two pathways of the EASI model have been explored in a scattered and non-cumulative way**, with a focus on the affective pathway as enabling and on the cognitive pathway as a suppressing mechanism. There is only one study that explores cognitive variables as facilitating mediators, and there are no studies about the suppressing role of affective variables.

There is also some evidence that **emoji effects are treated as social phenomena** (20%), for example, by employing mediators such as affective social presence, perceived sincerity, and willingness to forgive.

What is notable in the analysis of the mediators is the **absence of variables central to the most prominent theories, indicating an isolation from theory**. Also, there is very limited focus on attention, comprehension, and memory as variables that could **mediate** the effect of emojis on user engagement.

Taken together, the analysis of moderating and mediating variables reveals a field where more than half of the studies do not explore moderating or mediating relationships and the other half is scattered across many different variables without replication and mostly consistent with basic factors such as emoji presence or product type.

#### 4.3.5. Emoji Frequency and Emoji Combinations

Our analysis of the emoji frequency (how many emojis are tested), where it was available (n = 13), uncovered that most studies either use a single emoji (53,85%) or multiple emojis at the same time (23.08%). While frequency has received extremely limited attention from researchers so far, it seems that more emojis could hurt the perceived competence of the brand but improve the trustworthiness of reviews (Orazi et al., 2023; Huang et al., 2021).

A contradictory finding is that users find emoji combinations, especially rare ones, very interesting (Guo & Wang, 2024; X. Wang et al., 2023b). These findings are puzzling, given that two of these studies are for the same product category (accommodation). A possible explanation for the discrepancy lies in the methodological choices, with the study of Orazi et al. employing large scale field data and experiments, while Guo and Wang had a qualitative approach.

#### 4.3.6. Emoji Placement

Regarding placement, presented in Table 8, **over half of the studies that report where the emoji was placed have put it at the end of the sentence**. In fact, there was not a single case where the emoji was used at the beginning of the phrase, and the question remains whether findings about the effectiveness of emoji presence and emoji frequency would be valid if the emojis were placed at the beginning or even in the middle of the sentence.

Previous results reported in linguistics research show there is a semantic benefit when emojis are sentence-final (Grosz et al., 2023), but at the beginning of the phrase, they could serve as emotional primers (Dai et al., 2024).

Moreover, while previous studies generally agreed that emojis are better processed when they act as a supplement than a replacement of words, in our corpus of studies, there is a case (C. H. Kim et al., 2024) with contradicting evidence showing that artifact emojis worked better when they replaced words; otherwise, they increased skepticism. Finally, while **almost all studies used the emoji alongside the text**, there was one case with a different approach, where the emoji was used in a video and as a subliminal cue.

#### 4.3.7. Emoji Sentiment

Despite the primary focus of emojis as emotional cues, few studies (n = 11) reported the selected emoji’s sentiment (Table 9). There is **a clear positivity bias** explained by the focus on user engagement and purchase intentions and by a culture bias or a publication bias. The underlying reasons could be rooted in an industry practice that promotes positive content (Pınarbaşı & Kırçova, 2021; Casado-Molina et al., 2022).

The only study experimenting with positive and negative sentiments, in the context of service recovery, has found that negative emojis bring higher satisfaction and repurchasing behavior (Ma & Wang, 2021), giving preliminary insights against the positivity bias and **opening the door to research in negative circumstances**.

#### 4.3.8. Emoji Type and Function

The emoji type (face, objects, etc.) was recorded whenever it was available and is presented below in Table 10. **The most frequent emoji type used in research is by far the facial emojis,** with an impressive 62% testing only facial emojis. It seems that **emoji research has still not recognized the diversity of emojis**.

Facial emojis, due to their resemblance to human faces, have human properties and could also be used as priming cues, as we are wired to detect faces (Weiß et al., 2020). Similarly, they are perceived differently in the text compared to non-facial emojis (Cao et al., 2024), and while they can both carry emotions, we have not yet uncovered the dynamics (Riordan, 2017b). A question remains as to whether we can extend the findings on non-facial emojis, especially the ones about affect. Would the EASI theory be valid for non-facial emojis? Implications are discussed in the future directions section.

**Dominance of the emotional expression function** is observed. Two shortcomings are that, first, there were no studies exploring only the semantic aspects of emojis (i.e., their role in enhancing the meaning of the text) or the navigational/emphasis/decoration aspects, and second, the limited sample of comparative studies constrains the picture we have so far. Results are presented in Table 11. This finding reveals a **potential anthropomorphic bias**.

Data about worldwide emoji usage shows a significant gap between research and practice, with popular non-facial emojis being underrepresented in marketing research. (Unicode Consortium, n.d.; Emojipedia, n.d.).

How is this gap explained? Since data about emoji usage do not discern between brand or consumer use, a possible explanation is that brands do not necessarily follow the same patterns in emoji usage as users, and this is then represented in emoji research. Additional data about emoji usage patterns from brands—and a comparison with user data—is needed, but there is some initial evidence that the above assumption is true (Pınarbaşı & Kırçova, 2021; Casado-Molina et al., 2022).

Additional research would uncover if brands use facial emojis more (in line with researchers’ choices) and if emotional expression is the primary reason why emojis are used in brand communications.

The key findings of the above analysis of characteristics are presented in the Appendix A, Table A14, alongside implications for future studies.

### 4.4. Methodology (M)

#### 4.4.1. Category of Methodological Designs

The analysis of the methodological choices in Table 12 shows that **the field has favored an experimental approach** to test the effects of emojis on specific consumer behavior dimensions. The use of mixed methods shows a more refined approach combining multiple studies to better understand emojis with triangulation. The above findings indicate, first, that research on emoji usage in a digital marketing and advertising context is still in its infancy, and second, effort has been put into understanding if results from psychological and linguistic studies about emojis can be linked to their marketing effectiveness. Lastly, the presence of only one longitudinal study potentially means that emojis are perceived by researchers as having only short-term implications. These observations, together with the limited use of survey and questionnaire studies, suggest that we mostly try to understand how consumers respond to them but not “why” they respond in a particular way.

#### 4.4.2. Sub-Categories of Methodological Designs

The sub-categories for the three dominant categories are presented in the Appendix A, Table A10, Table A11 and Table A12. The emphasis on online experiments (30,8%) and lab experiments (23,1%) and the limited use of complex approaches suggests that **emoji research in marketing is still in its formative years and suggests opportunities for more refined designs, where qualitative studies explore emoji perceptions and then quantitative studies test the findings**.

For content analysis, researchers seem to prefer human over automated analysis, probably because of the subtle nuances and the multiple roles of emojis (semantic, symbolic, and emotional), or because there are not enough classification systems or theoretical frameworks for categorizing emoji usage.

The notable **underrepresentation of field experiments** and their combination with other experiments in mixed studies show that they are mostly used as confirmatory methods.

In sum, **the analysis of the methodological choices suggests fragmentation**. On the positive side, the field promotes controlled experimental designs to uncover causal relationships and mixed methods to triangulate and build robust results.

Some concerning patterns also emerge. The limited focus on survey studies, together with a minimal presence of qualitative studies, shows a low interest in understanding the “why” behind the effectiveness of emojis, and the minimal presence of longitudinal studies shows an emphasis on the immediate returns rather than on the sustained effects.

#### 4.4.3. Sample Characteristics

Analyzing the sample of experimental studies, we found several limitations that constrain the generalizability of the findings. While the most common cluster is around 100–400 participants, sample sizes varied drastically (Appendix A, Table A13) because of the different experimental procedures, resulting in inconsistent findings.

We notice a **bias toward younger participants** (18–39), with several studies only working with Generation Z. The reason may be that they are heavy users of emojis, or the emphasis is a result of convenience samples, as researchers are working with university students who are easier to be recruited (Ashraf & Merunka, 2016; Vinson & Lundstrom, 1978; Jones & Sonner, 2001; Fuchs & Sarstedt, 2010). The need for multiple samples has been recorded on many occasions (Espinosa & Ortinau, 2016), and it is crucial to focus on other generations, as emojis can be interpreted in different ways by different generations.

The vast majority of designs relied on binary gender categories and had mostly female samples. Only one study (Neel et al., 2023) explicitly included LGBTQ+ participants, showing that there is space for more inclusive sampling.

All the above patterns collectively indicate a field that needs theory development, methodological sophistication, and a deeper understanding of the mechanisms underlying the relationships of emojis with consumer behavior variables.

## 5. Discussion and Future Research Agenda

**Scheme 3:**
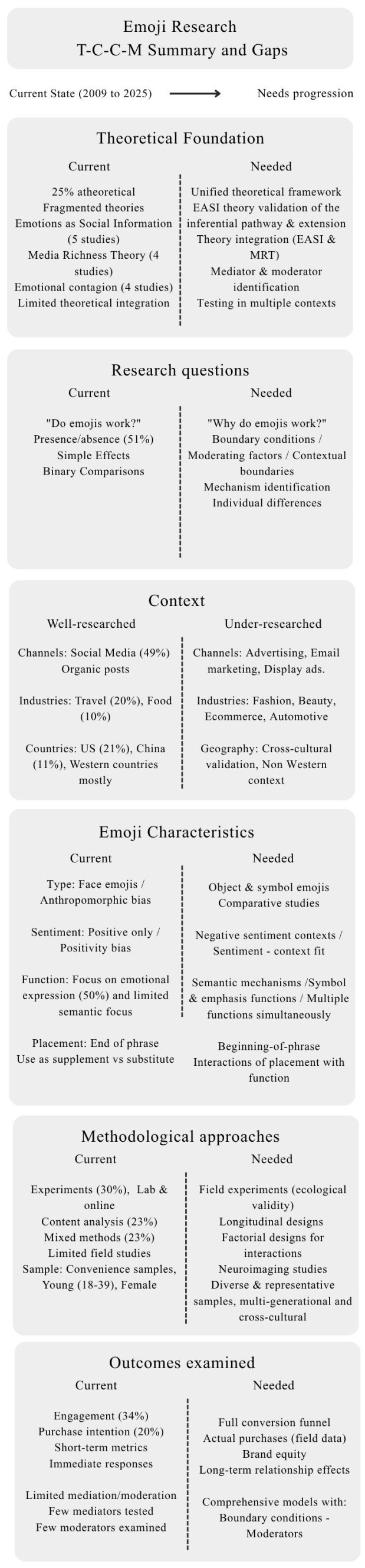
Visual summary of the current state of research and future directions. Image created by authors in Canva.

In an effort to create a more comprehensive view of the TCCM findings, we created a framework depicting the current state of the field and the direction of future studies. It is presented in Scheme 3.

### 5.1. Theory Development

Our analysis revealed some theoretical gaps that are acknowledged in this section. With nearly 25% of studies lacking theoretical grounding and with 52 explicit theories identified in 46 articles, the need for theoretical integration is evident. Our most compelling finding here is the Implicit Theory revealing the hidden theories of atheoretical studies. Based on the analysis, we then propose a theoretical integration.

While the EASI theory and emotional contagion theory have received the most attention from researchers, taking into account both explicit and implicit theories, our suggestion is to **test the EASI theory in more design settings and with the intention to discover how the two mechanisms (inferential or affective) work for emojis**. The reason is that the emotional contagion theory works more in automated, primitive ways and cannot explain why emojis bring the desired effects.

Given the focus of studies on how the presence of emojis is linked to consumer behavior, the choice of the emotional contagion was justified, but in the future, where more complicated research hypotheses must be tested, we posit that the emotional contagion theory is not enough to explain the mechanisms under which emojis work.

Given that in some cases we found contradictory findings, the EASI theory would be beneficial to address gaps. We would like to explain two indicative cases. First, from our analysis of dependent variables (see Section 4.3.2), we noticed the contradicting results of emojis when it comes to Purchase-Related Outcomes for the same product in different contexts (lab experiment and field experiment in Facebook ads) (Das et al., 2019; Lee et al., 2021). Under the EASI theory, the discrepancy would be explained by the perceived appropriateness, a core variable that has been found to serve as moderator in EASI (Van Kleef, 2009). Second, in the study of Huang et al. (2021), emojis had mixed results on purchase intention of gifts for different relationships. Relationships would be the moderator of the perceived appropriateness and of the relative strength of the pathways.

Upon reflection of the examined studies and more, the questions remaining are as follows: Is the EASI theory valid for non-face emojis that convey emotions (e.g., hearts)? Can the EASI theory be used to explain actual outcomes such as user engagement and purchases? How does the product type or the product message interplay with the affective and inferential pathways? What other model can explain the use of emojis in marketing and advertising, especially when there is no emotion conveyed (e.g., in pure symbols)? Regarding the emotional contagion theory, the largely automatic process implied leaves the mechanisms vague and, thus, are not easily tested.

Media Richness Theory could be used in other settings beyond content analysis. While the start may be to compare no-emoji versus emoji conditions, since emojis can enhance the text in various ways (symbolic, emphasis, semantic, and emotional), **more complex designs are necessary to define richness for emojis**, measure it, and explain which properties of the emoji usage add richness. Under MRT, emojis make communication rich and facilitate processing. However, other research (Barach et al., 2021) has found contradicting evidence that emojis do not always bring faster processing, and semantic congruence has to be carefully examined.

The role of culture has received minimal attention so far. This is a clear research gap that could be addressed by future studies, especially because Western, individualistic countries are commonly used for emoji research. While there is evidence that emojis, similarly to basic emotional expressions, may be interpreted universally across cultures, there are also cases where cultural dimensions influence consumers in complex ways (Neel et al., 2023; P. Wang & McCarthy, 2020).

Upon reflection on the above-mentioned theories, we found it fascinating that **the notion of ambiguity, central to MRT and EASI, was also found in the implicit theories analysis**. Ambiguity could also be linked conceptually to Hofstede’s dimension of “uncertainty avoidance”. Taken together, we have a beautiful theoretical convergence around ambiguity that could drive more integrated research on questions such as the following: (1) How do brands in high versus low uncertainty avoidance cultures differing in their emoji use? (2) When using emojis in marketing, how do we define ambiguity? Are there different types, such as emotional/semantic ambiguity, that reflect the multiple roles of emojis? (3) Which emojis are the least ambiguous across cultures? (4) How and when do emojis reduce (or increase) the ambiguity of the marketing message? (5) How can the emotional contagion theory, which relies more on an automated process, be used to explain the role of emojis in ambiguity?

As some of the aforementioned theories complement each other and some are completely different in their approach, we created Table 13 to map theories involved in our corpus of studies, how they contribute to emoji research, and their limitations.

We propose EASI as the main framework in emoji research. Because of its dual pathways that match the two main functions of emojis, emotional and semantic, it has the potential to explain emojis, and, thus, we explore the possibilities of its integration with other theories in the same table below. Future research could test these possibilities.

### 5.2. Context

Regarding the context of future studies, our analysis suggests that there are opportunities for a deeper look in more industries, marketing channels, platforms, and cultural settings.

#### 5.2.1. Industries and Product Type

As research about emojis progresses, the underrepresented industries (fashion, beauty, e-commerce, etc.) and the industries not appearing in our sample (automotive, services like insurance/telecommunications/energy, fitness/wellness, healthcare, and pharma) could be further explored to understand if emoji effects are universal. The product type (hedonic or utilitarian) may be a moderator, as found in the work of Das et al. (2019) and Mladenović et al. (2022).

#### 5.2.2. Marketing Channels and Underrepresentation of Advertising

The observed effects of emojis can be studied in more marketing channels and platforms with a more holistic approach. Results from studies located in an organic social media context should be examined in other settings, such as social media advertisements, email marketing, and display ads. By systematically comparing emojis across channels and platforms, we will be able to uncover moderators and mediators of the relationships. At a practical level, marketers will be able to tailor their strategies and make a more efficient use of emojis.

The absence of advertising research is also a critical gap that must be addressed. Exploring whether results from organic social media content are reproduced in advertising will be practically beneficial, and it will also shed light on our previous theoretical suggestion that EASI is the right theoretical basis to explain emojis in marketing. The main reason is that, in advertising, message types can be drastically different than in organic content, in that the content is more persuasive and potentially assertive. **Emojis in advertising could potentially trigger psychological reactance of the users because they see the message with more skepticism than organic posts**, which may be perceived as more authentic and informal.

Linking this point with the theoretical discussion and the emergence of EASI as an appropriate framework, the concept of perceived appropriateness under the EASI theory (Van Kleef et al., 2011) and the concept of persuasion knowledge (Friestad & Wright, 1994) could be relevant here. If reactance happens because of the awareness of the ad, it could affect both the inferential and the affective pathways: the first because the emotion would seem fake and the second because of the negative reaction (Evans & Park, 2015). This may be tested with designs exploring the same emoji characteristics in two settings: organic posts and social media advertising.

On the other hand, could emojis reduce psychological reactance in advertising? Taken together, our previous findings about the informal nature of emojis, their connection to brand Anthropomorphism, and the fact that they carry emotions and are more conversational because of their everyday use in messaging apps, show that they could actually reduce resistance to advertising by minimizing the sense that the message is corporate or assertive.

Customers are becoming more aware of covert advertising practices, and perceived covertness, intrusiveness, and manipulation have been linked with negative attitudes to the ad (H. Kim et al., 2025). In this direction, **emojis could signal friendliness and authenticity, and they could mitigate the negative effect**. Linking back to the EASI theory and perceived appropriateness, if emojis are perceived as a genuine attempt by the brand to communicate in a warm and friendly way, then they are beneficial. While there are no studies exploring this question, humor in advertising has been shown to reduce resistance under many conditions (H. Kim et al., 2025).

Given the initial emphasis on Purchase-Related Outcomes, advertising would be the ideal channel to explore these dependent variables with ecological validity, as it relies on conversion metrics. We acknowledge the multitude of obstacles faced by researchers (budget, access to advertising accounts, and lack of consent to use business data) and we propose another route to overcome them. Researchers could create partnerships with advertisers or agencies to conduct A/B tests in real campaigns and to reveal if findings about emoji effectiveness translate to actual advertising performance.

#### 5.2.3. Cross-Cultural Examination

Cross-cultural studies about emojis remain underexplored and can be addressed in the future. Given the analysis, at the moment, **we cannot assume universality or cultural specificity for emoji characteristics**. Future research should systemize cross-cultural comparisons and address the significant geographic imbalances. A good start would be to address if and how cultural dimensions moderate the two pathways (inferential and affective) of the EASI theory. There is some evidence that East Asians and Western people are not influenced by faces in the same way (Fang et al., 2021), and this could be validated for facial emojis, too.

Also, the individualism/collectivism of the culture could be of importance in emoji usage, so differences in emoji usage by brands and the perceptions of consumers may be noticed (Togans et al., 2021).

Moreover, since ambiguity has emerged as a potential moderating factor, we posit that it may be tolerated differently in countries with high uncertainty avoidance; thus, it may be tested in countries with low versus high scores.

### 5.3. Characteristics

The analysis of the characteristics of the studies reveals several significant opportunities for future research schemes. We draw on the findings and the future directions in the Appendix A, Table A14.

First, there is a **gap in understanding the antecedents of current emoji use by brands**. Studies that will try to understand why some firms do not use emojis in their strategies could further advance the field, given that most experimental designs use emojis as an independent variable.

Second, future studies should move beyond the basic structures of Emoji-to-Engagement or Emoji-to-Purchase Intention and **explore more complex approaches, firstly by including mediators** that would give a deeper view on the observed results and secondly by measuring actual purchases instead of intentions. If the emojis have so much power to affect purchase intentions, then we need to go “back in the lab” and understand why by looking at variables across the funnel.

Third, **more emoji attributes must be examined** (as independent variables or as moderators) to understand under which circumstances the presence of emojis or number of emojis have specific effects. The semantic congruence of emojis with the text may be very important. In this direction, we may need to apply cross-cultural designs, as some emojis probably carry different meanings in different cultures.

Fourth, something equally important is to see **if and how the surrounding text, platform features, and audience characteristics play a role in emoji effectiveness** and how they interact with other elements (e.g., the photo of the post or the advertisement).

Fifth, **time-related variables**, such as frequency over time (repeated exposure) or the time of day, **may also be incorporated in experimental designs** to uncover whether the effects are sustained. We have mentioned that the focus is on short-term results, but actually, this is implied by the choice of dependent variables, as time has not been discussed at all as a variable. Our suggestion is based on previous research about differences in arousal and valence at various times of the day (English & Carstensen, 2014), diminishing results of persuasion attempts over time (Vardikou et al., 2025a), and the effects of repetition (Schmidt & Eisend, 2015).

Sixth, we would expect to see **more studies examining the congruence of the emoji with brand traits**, as less emphasis has been given on the relationship of emojis with attitudes about the brand (as a dependent variable) and brand characteristics (as independent variables).

Seventh, **emoji frequency in content (how many emojis are present/tested) and combinations should be reported better** in the next studies, as it may affect results, and, ideally, it should be tested to understand more about the optimal use of emojis in the text.

Eighth, since “position might be an important factor in emoji processing” (Tang et al., 2024, p. 8), the choice of **placement of emojis must be carefully established and reported**, as so far it seems that processing fluency but not comprehension is hurt by the substitution of words with emojis (Orazi et al., 2023; Scheffler et al., 2022).

Ninth, it would be crucial **to test the effects of emojis in settings where negative sentiment is conveyed, for example,** in urgency/scarcity messages, cause-related content, or sustainability messaging. Until now, we observe a positivity bias, while emojis with a negative sentiment are widely used (Pınarbaşı & Kırçova, 2021; Casado-Molina et al., 2022; Unicode Consortium, n.d.), Before that, though, we need more robust evidence on the question of whether brands follow the same patterns of emoji usage as users.

Tenth, **a wider spectrum of research using object and symbol emojis or comparing facial emojis with the other types is necessary**. The interplay between the emoji type (face, object, and symbol) and the semantic and emotional congruence of the emojis may also be of importance, since both face and non-face emojis carry meaning and emotions. Initial evidence suggests that the use of the same “common” emojis may not be beneficial to brands, as they are “uninteresting” (Guo & Wang, 2024).

### 5.4. Methodology

According to our methodological analysis, future emoji marketing research needs to follow multiple essential directions, which will improve both theoretical knowledge and practical implementation.

There is a current focus on online and lab experiments, on manual content analysis and on mixed methods, which shows that the field is emerging. In addition, we only explore what is working in the short-term and how firms use emojis to increase engagement.

Therefore, the field could progress to a stage where (1) we study how emojis interact with different contexts and consumer characteristics; (2) longitudinal research is employed to **study effects over time**; (3) field experiments need to become the primary research method for **evaluating emoji effectiveness with ecological validity**; (4) qualitative emoji perception studies will guide subsequent quantitative assessments; (5) the development of automated content analysis tools together with standardized taxonomies could **enhance manual coding methods**; (6) neurophysiological tools in neuromarketing studies are used to test emoji effects that cannot be observed in other ways, variables that precede engagement, and the informational and emotional processing of emojis; and (7) sampling must have diverse demographics and cultures to represent the global and multigenerational nature of emoji communication.

Regarding **gender**, since there is evidence of different comprehension of emojis for males and females (Dalle Nogare et al., 2023; Prada et al., 2018), future studies should incorporate it in their designs.

The implementation of these methodological improvements will advance emoji marketing research from its present state of infancy into a strong theoretical framework with practical applications.

## 6. Theoretical Contribution

This domain-based systematic literature review makes several theoretical contributions towards understanding emojis in a marketing context. With a T-C-C-M approach, we analyzed the current state of the field and the research gaps that may be addressed by future studies.

The analysis of explicit and implicit theories shows that (1) emojis in marketing are mostly researched with grounding in psychological theories, (2) there is a large body of studies that are atheoretical, and (3) many diverse theories are used.

All the above show that we need more theory-driven research that will be based on the mentioned theories and a robust theoretical framework to explain what kind of relationship the emojis have with consumer behavior variables as outcomes and under which circumstances.

This is the first systematic review to uncover implicit theories in emoji research. We revealed seven theories that were subtly used as the foundation, and these included Anthropomorphism, Communication Accommodation Theory, and emotional contagion. With this implicit concept analysis, we also found that emotional contagion is the most commonly used theory, together with EASI, and this result further means that the analysis of implicit theoretical concepts should be included in systematic literature reviews, as it may completely alter the landscape.

Our Implicit Theory analysis identified ambiguity as an important concept in emoji research that should be incorporated in future designs. The definition and measurement of emoji ambiguity are critical steps in this direction.

Under the TCCM framework, we uniquely identified other characteristics of emojis, and we suggest that a more robust theoretical framework is created—one that unifies all the properties of emojis, such as semantic, emotional, symbolic, and decorative. The EASI theory could cover the semantic and emotional properties, but there was only one attempt to extend it to emojis (Erle et al., 2022), which only confirmed that, for emojis, the affective pathway works in the same manner.

## 7. Managerial Implications

Emojis have been examined for their power to influence user engagement and purchase intentions, and marketers may find rich information on settings under which emojis are effective to drive immediate outcomes as well as repurchase intentions.

The observed results are mostly owed to the emotional aspects of emojis and are linked to brand Anthropomorphism that helps brands obtain a human-like representation in their consumers’ minds. So far, evidence shows that the presence of facial emojis of a positive sentiment is beneficial to brands—especially the ones with hedonic products—as **they are better processed when they complement (and not replace) the word and they are mostly placed at the end of the sentence**. When designing more complex combinations of text and emojis (i.e., multiple emojis in long texts), marketers may experiment.

To answer the question “how to better use emojis in marketing communications”, it seems that “less is more”, and the optimal quantity is up to three emojis (Manningham et al., 2024). However, this finding has to be tested more for more complex combinations of text. Another finding is that emojis facilitate the processing of the text when they supplement it rather than when they act as substitutes for it (Maiberger et al., 2024; Orazi et al., 2023). Regarding placement, research has favored the end-of-sentence position, so marketers should further test if emojis at the beginning of the phrase would serve as emotional primers.

The combined limitations of (a) the absence of a unified theoretical framework, (b) focus on the travel and food industry, and (c) limited attention on the antecedents of emoji usage by brands and of their interplay with brand traits (beyond trust and competence) suggest that marketers should include experimentation with emojis in their strategies so that they understand when emojis work for their brands. The limited exploration of emojis in the marketing of other industries (fashion, automotive, and e-commerce) means that, in these industries, marketers who experiment with the optimization of emoji usage have the advantage of the “first move”.

Regarding the conditions under which emojis are effective, it seems that context matters, and product type, platform, and location must be taken into account. First, for hedonic and experiential products, such as in the travel and food industry, emojis work particularly well. For utilitarian products, there is limited research, but this finding creates opportunities for pioneering brands that will experiment with emoji usage. Second, since most emoji research is in organic social media content (and mostly from Twitter/X, Facebook, and Instagram) and less attention has been given to other contexts (advertising, email marketing), marketers have to recognize that effectiveness may vary on different platforms and even in different placements on the same platform (e.g., Instagram reels). For email marketing, marketers have the opportunity to test emojis directly inside their email marketing software, either in the subject line or in the body of the email. The same applies to social media advertising, where platforms usually offer an a/b testing tool. In sum, marketers can experiment with emojis more to understand if they are appropriate and effective for their brand.

Primary evidence has shown that emojis have an interplay with the source of the message and with the relationship with the brand. Since long-term results have not been examined in any of the studies, additional attention must be given by marketers to ensure that emojis are effective in the long-term or at least do not do damage.

Given the limited focus on emojis of a negative sentiment, marketers should use emojis with caution in sensitive contexts, such as crisis communication and for serious issues and topics. Since the complexity and ambiguity of emojis have emerged as core topics but are not examined in multiple contexts, marketers could stay on the “safe” side by choosing emojis which are not ambiguous. Understanding of how emojis interact with different message types or brand characteristics is limited; thus, marketers could use emotional emojis for engagement but refrain from using them for more serious informational topics.

This advice is particularly important for global brands, as there is little evidence from multi-cultural perspectives. Regarding culture appropriateness, since the vast body of research has been conducted in the US and China, we cannot assume the universality of results, and emojis have to be locally tested. Global brands also have the opportunity to experiment with emojis in order to understand if they work in a universal way for their brand or to inform their marketing strategy by adapting a “glocal” communication strategy. Similarly, because there was a preference by researchers for young female respondents, marketers targeting other audiences have to be even more proactive to validate findings.

Our study revealed that emoji research is still at a foundational stage, and the absence of a unified theoretical framework means that we rely mostly on empirical data, and we cannot truly answer “why” and “how” emojis are effective in marketing communication. Our suggestion for marketers is to adopt an experimental mindset and to consider fit with their brand, target population, and message type.

## 8. Limitations

Several limitations must be acknowledged in our methodology and results to properly guide future research. The most significant limitation of our study comes from the choice to uncover implicit theoretical concepts. Despite our effort to systemize the process by coding with the Gioia method and by using bracketing to leave aside our biases that could result from the analysis of the explicit theories, subjectivity can never be fully eliminated, as researchers always have a specific point of view (Thompson et al., 1989). We acknowledge that other researchers could find different ways to approach the implicit theoretical concepts and potentially produce different results with their analysis. Responding to the reviewers’ comments, we took additional steps of triangulation of implicit theories with theories used by the same authors and by articles citing the study of the corpus. However, an additional step towards a more robust analysis would be to employ experts from other fields (e.g., psychology).

The choice to apply the T-C-C-M framework comes with robustness but also with limitations, as it may limit the understanding of the connections between the four aspects. We recognized this limitation from the beginning, and we made attempts to address it by synthesizing knowledge across aspects whenever possible.

Our deliberate choice to include only papers published in peer-reviewed journals potentially excluded cutting-edge articles that were published in conferences or other journals. Given the fast-growing nature of emojis and digital marketing, and the higher volume and speed of conference publications compared to the lengthy process of the journals, we may have missed some recent developments in emoji research. In addition, our -previously justified reliance on Scopus for the initial dataset may have restrained the sample by missing articles that were only indexed by Google Scholar or Web of Science.

Similarly, the choice to include only English-written articles may have introduced a cultural bias, and thus, not only should aspects of the analysis that concern culture and countries be treated with caution but we have also limited our understanding in how emojis are researched in other languages.

We also acknowledge that this is a static analysis and early findings about emojis may now be obsolete due to the fast-paced nature of emojis.

## 9. Conclusions

We began this review with the question “how are emojis researched in marketing and advertising” and we found that, despite the omnipresent nature of emojis, it is **a growing yet underexplored field**, with plurality in terms of theoretical frameworks used and methodologies employed, but with a concentration of research on facial emojis, on their emotional expression (rather than the semantic and symbolic properties) with a clear focus on the positive sentiment, and on their use in social media posts. Interestingly, while the commercial focus is apparent, little attention has been given to the use of emojis in online advertising and e-commerce, showing a clear research gap.

Our analysis establishes that current emoji research operates at a foundational level, prioritizing the basic question of “whether emojis work” rather than the more sophisticated questions of “why, how, and when emojis are effective.” We highlighted the need for a unified and robust theoretical framework and for more advanced methodological choices in terms of the characteristics of emojis beyond the mere presence of emojis, including mediators and moderators.

We present a visual analysis for the key theories with recommendations on how three of them could be nested under EASI. If EASI is chosen as the most appropriate theory, both its inferential and affective pathways have to be explored under different designs, and the perceived appropriateness of EASI may be linked to context-fit, brand-fit, and message-type-fit. Ambiguity, a notion common in the dominant theories, has to be central in future studies.

Regarding the context of future studies, our analysis showed that cross-cultural settings and the geographical breadth could be sought, and there is a need to cover more industries, with both hedonic and utilitarian products, that were underrepresented. These include fashion, beauty, and e-commerce together with traditional sectors such as automotive, insurance, and emerging sectors such as fitness and wellness. Marketing channel differences and platform differences may be examined, as well as multi-channel approaches. As the current focus was on organic social media content on Twitter and Facebook, only a few cases focused on advertising and e-mail marketing, as well as on TikTok, YouTube, and other platforms. This progress will further uncover moderators and mediators. At a practical level, it will guide the decisions of marketers towards a more efficient use of emojis.

Several opportunities emerged from the analysis of the characteristics of the studies that showed a focus on studying the effects of emojis, while the antecedents of emoji usage by brands are slightly explored. Since the primary focus of studies is the relationship of the presence of emojis with engagement and purchase intention, future studies could explore more complex designs in which aspects of emojis are studied and mediators and moderators are employed. These choices could be combined with the EASI theory to understand which factors influence the affective and the conscious/inferential path.

Methodologically, there is a wide range of methodologies used, but the field could progress with factorial designs that will study the boundary conditions and the mediators that play a role in the effectiveness of emojis in engagement and purchase intention. In that direction, and since the focus on commercial results is strong, field experiments will show if emojis have the power to affect actual sales. We also posit that the use of neurophysiological and neuroimaging tools may be used to directly test variables that precede engagement, such as attention and emotion. Designing studies beyond the convenient samples would mean having diverse demographics and cultures. A question that still remains is whether emojis can be used universally in the marketing communications of global brands.

Our analysis has managerial implications for marketers and advertisers. While positive facial emojis are generally associated with improved results, we do not know the effects of negative emojis or of other emoji categories beyond faces. There is evidence that emojis could affect the perceptions of customers, engagement, and purchase intentions, but given the absence of a unified theoretical framework, marketers are encouraged to take an experimental approach to the use of emojis.

## 10. Final Reflection

We have found that research on emojis in marketing and advertising is in an early and growing stage, and we provided suggestions for future research grounded in our analysis of the studies. However, we need to acknowledge that their ever-evolving nature, as they are a “living” paralanguage, will make research more challenging. We are aware that, because of their extremely frequent use in texts produced by artificial intelligence (what users describe as “emoji overload”), humans have started altering their preferences in regards to emoji frequency and the emoji symbols used so that they do not match A.I. patterns. All of the above construct a very dynamic and fast-paced environment, and future research needs to be adaptable and versatile.

## Figures and Tables

**Table 1:** Top 10 most cited articles in emoji research in marketing and advertising.

Title	Authors	Year of Publication	Number of Citations
Service with emoticons: How customers interpret employee use of emoticons in online service encounters	X., Li, Xueni; K.W., Chan, Kimmy Wa; S., Kim, Sara	2019	211
The magic of danmaku: A social interaction perspective of gift sending on live streaming platforms	J., Zhou, Jilei; J., Zhou, Jing; Y., Ding, Ying; H., Wang, Hansheng	2019	211
Emoji rhetoric: a social media influencer perspective	J., Ge-Stadnyk, Jing; U., Gretzel, Ulrike	2018	210
Textual paralanguage and its implications for marketing communications	A.W., Luangrath, Andrea Webb; J., Peck, Joann; V.A., Barger, Victor A.	2017	164
To emoji or not to emoji? Examining the influence of emoji on consumer reactions to advertising	G., Das, Gopal; H.J., Wiener, Hillary J.D.; I., Kareklas, Ioannis	2019	159
Lexicon-enhanced sentiment analysis framework using rule-based classification scheme	M.Z., Asghar, Muhammad Zubair; A., Khan, Aurangzeb; S., Ahmad, Shakeel; M., Qasim, Maria; I.A., Khan, Imran Ali	2017	149
Making food labels social: The impact of colour of nutritional labels and injunctive norms on perceptions and choice of snack foods	M., Vasiljevic, Milica; R., Pechey, Rachel; T.M., Marteau, Theresa Mary	2015	78
Service with a smiley face: Emojional contagion in digitally mediated relationships	L.W., Smith, Leah Warfield; R.L., Rose, Randall Lee	2020	71
Neuromarketing, subliminal advertising, and hotel selection: An EEG study	L., Hsu, Liwei; Y.J., Chen, Yen Jung	2020	68
Sex differences in emoji use, familiarity, and valence	L.L., Jones, Lara L.; L.H., Wurm, Lee H.; G.A., Norville, Gregory A.; K.L., Mullins, Katie L.	2020	66
Total number of citations for the top 10 articles			1387
Total citations for 140 articles			2780

**Table 2:** Top 17 sources with the highest article production.

Source Title	Number of Articles
Journal of Business Research	5
Journal of Research in Interactive Marketing	4
International Journal of Research in Marketing	3
Sustainability (Switzerland)	3
Applied Marketing Analytics	2
Australasian Marketing Journal	2
Computers in Human Behavior	2
Indian Journal of Marketing	2
Journal of Advertising	2
Journal of Consumer Behaviour	2
Journal of Destination Marketing and Management	2
Journal of Interactive Marketing	2
Journal of Product and Brand Management	2
Journal of Retailing and Consumer Services	2
Journal of the Academy of Marketing Science	2
PLOS ONE	2
Psychology and Marketing	2

**Table 3:** Countries’ citations. Top 20 countries are presented. Source: Authors.

Country	Total Citations	Number of Articles	Avg Citations
USA	937	33	28.39
China	457	22	20.77
India	350	19	18.42
Hong Kong	224	3	74.67
United Kingdom	203	7	29
Saudi Arabia	184	3	61.33
Taiwan	154	7	22
Pakistan	151	1	151
Australia	143	6	23.83
South Korea	90	9	10
France	84	4	21
Spain	78	8	9.75
Portugal	70	3	23.33
Poland	56	2	28
Turkey	37	2	18.5
Tunisia	37	2	18.5
Czech Republic	35	2	17.50
Netherlands	31	4	7.75
Brazil	29	3	9.67
Italy	28	1	28

**Table 4:** Explicit theories (n = 52).

Theory	No. of Times Appeared	Sample Articles
Emotions as Social Information Theory (EASI)	5	(Lee et al., 2021; Smith & Rose, 2020)
Media Richness Theory (MRT)	4	(Moussa, 2019; X. Wang et al., 2023b)
Hofstede’s Dimensions	2	(C. H. Kim et al., 2024)
Emotional Contagion	2	(Das et al., 2019)
Other Theories ^1^	29	
No Guiding Theories ^2^	10	

^1^ Other theories are the theories that have occurred less than two times in the literature dataset. ^2^ No guiding theories indicate the number of research articles that do not mention the use of any specific theory.

**Table 5:** Marketing channels of experiments/analysis (n = 43).

Channel	Number of Articles
Social Media	21
Not Applicable	6
Social Media Influencer	4
Messaging	3
Social Media Ads	2
Advertising	2
Website/Ecommerce Platform	2
Email Marketing	1
Online Video Platforms	1
None	1

**Table 6:** Platforms of experiments/analysis (Total = 20).

Platform	Times Appeared
Twitter/X	6
Facebook	3
Instagram	3
Facebook/Instagram	2
Facebook Sponsored Ads	1
TikTok	1
YouTube	1
Kakaotalk	1
Sina Weibo	1
Airbnb	1

**Table 7:** Dependent variables—consolidated table (n = 35 studies).

Dependent Variables	Frequency
User Engagement/Customer Engagement	12
Purchase-Related Outcomes	7
Specialized Measures	5
Message and Communication Effects	4
User/Customer Evaluations	4
Customer Satisfaction and Loyalty	2
Not Applicable	1

**Table 8:** Placement of emojis (n = 23).

Emoji Placement	Number of Studies
End of phrase	9
Did not mention/not applicable	8
Supplement	2
Supplement/replacement	2
In the video	1
Replacement	1

**Table 9:** Emoji sentiment (n = 19).

Emoji Sentiment	Number of Studies
Did not mention/not applicable	8
Positive	7
Positive and negative	3
Neutral	1

**Table 10:** Emoji type (n = 19).

Emoji Type	Number of Studies
Face	8
Did not mention/not applicable	6
Face and object	2
All	1
Artifact emojis	1
Multiple	1

**Table 11:** Emoji functions studied (n = 20).

Emoji function	Number of Studies
Emotional expression	7
Did not mention/not applicable	6
Emotional expression and emphasis	2
Emotional expression, emphasis, and decoration	2
Category and sentiment	1
Emotional expression vs. decoration	1
Word replacement and emphasis	1

**Table 12:** Methodological categorization (n = 43).

Emoji Function	Number of Studies
Experimental Studies	13
Mixed Methods	10
Content/Text Analysis	10
Machine Learning/Data Mining	8
Survey/Questionnaire Studies	1
Behavioral Data Analysis	1

**Table 13:** Summary of key theories and their integration.

Theory	Where It Can Be Used	Limitations	Integration with EASI	Future Research Priority
Emotions as Social Information (EASI)	The dual pathways (inferential and affective) influence observers. Relevant for the emotional and semantic aspect of emojis.	1. Does not explain navigational aspects of emojis, 2. potentially not relevant for symbol and object emojis	-	High priority: Test both pathways for face and non-face emojis.
Media Richness Theory (MRT)	Platform characteristics/when emojis are necessary.	Cannot be used to explain effectiveness of emojis	It identifies the contexts and platforms where emojis are more suitable	High priority: Test in contexts with different richness.
Emotional Contagion	Automated affect transfer from the message source to the observer.	Does not explain cognitive/semantic aspects and information processing beyond emotion	It operationalizes the affective pathway (Herrando & Constantinides, 2021; Van Kleef & Côté, 2022)	Medium priority: Operationalize the affective pathway of EASI. Low priority: Test with repeated exposure.
Hofstede’s Cultural Dimensions	How emojis are interpreted and if they are effective in cultures that differ in the dimensions (e.g., individualism/collectivism, risk taking/uncertainty avoidance).	Cannot be used to explain the mechanisms under which emojis influence observers.	Culture as a moderator in EASI	High priority: Test how emojis influence observers of different cultures and, specifically, their inferential and affective pathways.

## Data Availability

All data created in this systematic literature review are presented in the body of the text and in the Appendix A.

## Abbreviations

The following abbreviations are used in this manuscript:

TCCM	Theory Context Characteristics Methodology
MRT	Media Richness Theory
EASI	Emotions as Social Information

## Appendix A

**Table A1:** List of articles (n = 43) included in the T-C-C-M analysis.

No.	Author(s)	Title	Journal
1	(Babalou et al., 2025)	Social media influencers as catalysts for transforming risk perception crafting destination image and elevating culinary tourism in Iran	Journal of Vacation Marketing
2	(Hotz-Behofsits et al., 2025)	Natural Affect DEtection (NADE): Using Emojis to Infer Emotions from Text	Journal of Marketing
3	(Myers et al., 2025)	Signals for Success: The Intersection of Influencer Linguistic Personality, Content, and Follower Size	Journal of Interactive Marketing
4	(Manningham et al., 2024)	Be Direct! Restaurant Social Media Posts to Drive Customer Engagement in Times of Crisis and Beyond	Tourism and Hospitality
5	(Cui et al., 2024)	Clicks for money: Predicting video views through a sentiment analysis of titles and thumbnails	Journal of Business Research
6	(Guo & Wang, 2024)	Finding the best creation strategy: How influencer’s photo-editing behavior affects consumer’s engagement intention	Journal of Retailing and Consumer Services
7	(Guzmán Ordóñez et al., 2024)	Analytical model to measure the effectiveness of content marketing on Twitter: the case of governorates in Colombia	Journal of Marketing Analytics
8	(Duffett & Maraule, 2024)	Customer engagement and intention to purchase attitudes of Generation Z consumers toward emojis in digital marketing communications	Young Consumers
9	(J. Yu et al., 2024)	Leveraging Emojis as Visual Semiotics for Enhanced Engagement in Destination Marketing	Journal of Destination Marketing & Management
10	(C. H. Kim et al., 2024)	When and why do artifact emojis lead to backfire effects on consumer response?	Journal of Consumer Behaviour
11	(Maiberger et al., 2024)	Let’s face it: When and how facial emojis increase the persuasiveness of electronic word of mouth	Journal of the Academy of Marketing Science
12	(Bombaij & Mokarram-Dorri, 2024)	Does Posting About Diversity and Inclusion (D&I) Improve Engagement in Social Media? Antecedents and Impact of D&I Communication Decisions	Journal of Interactive Marketing
13	(Einsle et al., 2024)	TikTok video formats’ impact on user interaction—evidence from the Ocean Race	Managing Sport and Leisure
14	(Madadi et al., 2024)	The semiotics of emojis in advertising: An integrated quantitative and qualitative examination of emotional versus functional ad dynamics	Psychology & Marketing
15	(X. Wang et al., 2023b)	When Texts Meet Emoji: A Multi-Stage Study of Tourism Brands	Journal of Travel Research
16	(Balaji et al., 2023)	Effectiveness of B2B social media marketing: The effect of message source and message content on social media engagement	Industrial Marketing Management
17	(Taba et al., 2023)	COVID-19 messages targeting young people on social media: content analysis of Australian health authority posts	Health Promotion International
18	(X. Wang et al., 2023a)	The interaction effect of emoji and social media content on consumer engagement: A mixed approach on peer-to-peer accommodation brands	Tourism Management
19	(Boman et al., 2023)	Strength in diversity: How incongruent racial cues enhance consumer preferences toward conservative brands	Journal of Business Research
20	(Neel et al., 2023)	Emoji Alter the Perception of Emotion in Affectively Neutral Text messages	Journal of Nonverbal Behavior
21	(Orazi et al., 2023)	Non face emojis in digital marketing: Effects, contingencies, and strategic recommendations	Journal of the Academy of Marketing Science
22	(Mladenović et al., 2022)	Emojis to conversion on social media.	International Journal of Consumer Studies
23	(Li et al., 2022)	The effect of emotion in thumbnails and titles of video clips on pre-roll advertising effectiveness	Journal of Business Research
24	(Indwar & Mishra, 2022)	Emojis: can it reduce post-purchase dissonance?	Journal of Strategic Marketing
25	(Hewage et al., 2021)	Consumer responses toward symmetric versus asymmetric facial expression emojis	Marketing Letters
26	(Huang et al., 2021)	An Emoji Is Worth a Thousand Words: The Influence of Face Emojis on Consumer Perceptions of User-Generated Reviews	Journal of Global Information Management
27	(Lee et al., 2021)	Investigating the Negative Effects of Emojis in Facebook Sponsored Ads for Establishing Sustainable Marketing in Social Media	Sustainability
28	(Yang & Shin, 2021)	The Influence of Shoppable Content Readability on Consumer Engagement in Brand Pages	Asia Pacific Journal of Information Systems
29	(Ma & Wang, 2021)	Smile or pity? Examine the impact of emoticon valence on customer satisfaction and purchase intention	Journal of Business Research
30	(Rúa-Hidalgo et al., 2021)	Understanding the Emotional Impact of GIFs on Instagram through Consumer Neuroscience	Behavioral Sciences
31	(Haltmayer & Gierl, 2021)	Emoji Your Story: The Advertising Effectiveness of Emoji-Based Narratives	Marketing: ZFP—Journal of Research and Management
32	(P. Wang & McCarthy, 2020)	What do people “like” on Facebook? Content marketing strategies used by retail bank brands in Australia and Singapore	Australasian Marketing Journal
33	(Hsu & Chen, 2020)	Neuromarketing, subliminal advertising, and hotel selection: An EEG study	Australasian Marketing Journal
34	(Moussa, 2020)	Complaining with emojis: Some conceptual and analytical aspects	Applied Marketing Analytics
35	(Nirmala & Babu, 2020)	Analytic-based product opinion detection algorithm for twitter microblogging network	International Journal of Communication Systems
36	(Smith & Rose, 2020)	Service with a smiley face: Emojional contagion in digitally mediated relationships	International Journal of Research in Marketing
37	(Romão et al., 2019)	Leveraging a luxury fashion brand through social media	European Research on Management and Business Economics
38	(Moussa, 2019)	An emoji-based metric for monitoring consumers’ emotions toward brands on social media	Marketing Intelligence & Planning
39	(Das et al., 2019)	To emoji or not to emoji? Examining the influence of emoji on consumer reactions to advertising	Journal of Business Research
40	(S. Yu et al., 2019)	Emoticon Analysis for Chinese Social Media and E-commerce: The AZEmo System	ACM Transactions on Management Information Systems
41	(Ge & Gretzel, 2018)	Emoji rhetoric: a social media influencer perspective	Journal of Marketing Management
42	(Yoo et al., 2018)	The Dual Processing of Donation Size in Cause-Related Marketing (CRM): The Moderating Roles of Construal Level and Emoticons	Sustainability
43	(Park & Sundar, 2015)	Can synchronicity and visual modality enhance social presence in mobile messaging?	Computers in Human Behavior

**Table A2:** Implicit theories.

Study Number	Extract	Concept (2nd Order)	Implicit Theory (Aggregated)
Study 1	“Facial expression emojis are emoticons designed to resemble human faces”	Anthropomorphism and Human Resemblance	Anthropomorphism and Social Presence Theory
Study 1	“...we focus on one defining feature of facial expression emojis—facial symmetry, and propose that… asymmetric emojis tend to elicit more favor able consumer evaluations”	Anthropomorphism and Human Resemblance	
Study 1	“Because they are perceived as more akin to human expressions”	Anthropomorphism and Human Resemblance	
Study 1	“People commonly see human faces in inanimate objects”	Anthropomorphism and Human Resemblance	
Study 1	“Consumers tend to anthropomorphize a car”	Anthropomorphism and Human Resemblance	
Study 1	“Because the capability of conveying human emotions is a main function sought in facial expression emojis, consumers are expected to ascribe more values and generate more favorable responses to asymmetric (vs. symmetric) emojis”	Emotion Expression and Reduction in Ambiguity	
Study 2	“Whilst we found the health departments frequently used emojis in their communication to young people”	Emojis are for Younger People/Generational Fit	Communication Accommodation Theory
Study 3	“Influence of Facebook’s ‘Positive Emoticons’ on ‘Instagram Likes’”	Platform-mediated Interaction Styles, Temporal Dynamics of Engagement	
Study 3	“Positive emoticons- likes, loves, aha and wow–, comments and shares on Facebook tend to increase as the number of likes on Instagram increases until it reaches a plateau”	Temporal Dynamics of Engagement	Computer-Mediated Communication and Network Effects
Study 3	“The user’s interaction on Instagram is almost instantaneous, generating a considerable amount of likes, whereas on Facebook users interaction is developed during a certain time... it is normal that when a user interacts with a Josefinas’ post, his/her friends will see it on their newsfeed, increasing the likelihood of the last ones also interacting with it”	Temporal Dynamics of Engagement	
Study 3	“Facebook encourages a more relational and not a reaction process and thus, the interactions are not immediate and fast as on Instagram, but they persist over time,”	Temporal Dynamics of Engagement	
Study 4	“Our approach utilizes the consumer’s positive, neutral, and negative emotions to ward content as a measurement of consumers’ engagement.”	Content Explicitness and Trust	Information Processing and Persuasion Theories
Study 4	“Several researchers have explored the direct negative effects of complexity on consumer engagement”	Content Complexity	
Study 4	“Sufficient explicitness of content is a measure of advertising quality—explicitness leads to trust in the brand”	Content Explicitness and Trust	
Study 4	“It can be predicted that commerce features with explicitness can be used to secure consumers’ trust of SNS brand pages”	Content Explicitness and Trust	
Study 4	“Reliability and convenience created by commerce features (i.e., shoppable tags) are the same factors that are degraded by content complexity”	Content Complexity	
Study 4	“The effect of emojis on polarity was not significant. Therefore, H2 that informality had positive effects on consumer engagement was rejected”	Emojis are Informal	
Study 4	“The effect of emojis—which is a detailed factor of informality”	Emojis are Informal	
Study 5	“Those tweets which do not exhibit any sentiment through word as well as emoticon are referred to as ‘neutral tweets’”	Emojis as Affective Indicators	Affective Science and Emotional Contagion
Study 5	“Unlike words, emoticons can convey the mood of the user with minimum ambiguity. Hence, emoticons are given a greater weightage than the corresponding words while mining sentiments”	Emojis as Affective Indicators	
Study 6	“Emoticon is a symbolic illustration of mind, mood, emotional state and feelings used by online community. Emoticons convey a message more effectively without having dependency on the language and specific domain”	Emojis as Affective Indicators	Affective Science and Emotional Contagion
Study 7	“Relative to text alone, messages embedding NF emojis increase (a) message evaluations and (b) eWOM volume”	Emoji Function (Complementary vs. Substitutive)	Signaling Theory and Source Credibility
Study 7	“The number of NF emojis moderates the effect of emoji function, such that, when emoji number is high (vs. low), substitutive NF emojis lead to (a) lower message evaluation and (b) decreased eWOM volume, compared to complementary NF emojis”	Moderation by Source Credibility (Seller Quality) and Quantity Effects (Emoji Overuse)	
Study 7	“Seller quality moderates the interactive effects between emoji function and emoji number on (a) message evaluations and (b) eWOM volume. For regular sellers, there will be a significant interaction effect between emoji function and emoji number. For premium sellers, the two way interaction effect will be attenuated such that using multiple emojis should have a negative effect irrespective of emoji function…will be mediated by perceived competence”		
Study 7	“To interpret in managerially relevant terms, complementary emoji presence in listing titles increases eWOM volume…”		
Study 7	“A three-way ANOVA revealed significant main effects of emoji function, emoji number, and seller quality on perceptions of sellers’ competence…no significant interactions for emoji function × seller quality or emoji number × seller quality but a significant emoji function × emoji number interaction”		
Study 8	“A new continuous variable is defined: the difference in the emotional valence between the implicit and the declared measures in each GIF (VD)”	Emojis as Affective Indicators	Emotional Contagion
Study 8	“This indicates that the greater the proportion of emojis used in comments, the greater the difference that will be found between the psychophysiological and the stated measure of valence”		
Study 9	“We focus on the content of the message (sentiment valence, use of emojis, and post vividness) and the features of the message (message length, use of hashtags, and use of mentions) as important characteristics of brand communication on social media”	Tone–Message Fit	Framing and Message Fit Theories
Study 9	“We argue that while emojis and vividness in messages can positively influence consumer attitudes toward brand communication, leading to increased consumer engagement, D&I communication calls for a more serious tone and central route of information processing, which consumers are likely to perceive as a better fit and appreciate more”		
Study 9	“Brands that use more emojis are less likely to communicate about D&I”		
Study 10	-	-	-

**Table A3:** Implicit theories triangulation.

Study Number	Implicit Theory (Aggregated)	Authors’ Publications Search	Citations Context	No. of Relevant Cited Articles	Implicit Theory (Corrected)
Study 1	Anthropomorphism and Social Presence Theory	Anthropomorphism	Anthropomorphism	2 out of 32	Anthropomorphism
Study 2	Communication Accommodation Theory	N/A	N/A	0 out of 25	Communication Accommodation Theory
Study 3	Computer-Mediated Communication	N/A	N/A	1 out of 81	Computer-Mediated Communication
Study 4	Information Processing and Persuasion Theories	N/A	N/A	1 out of 3	Information Processing and Persuasion Theories
Study 5	Affective Science and Emotional Contagion	N/A	N/A	0 out of 6	Affective Science and Emotional Contagion
Study 6	Affective Science and Emotional Contagion	N/A	Construal Level	2 out of 27	Affective Science and Construal Level
Study 7	Signaling Theory and Source Credibility	Multimodality	Multimodal Theory	2 out of 47	Multimodal Theory and Signaling Theory and Source Credibility
Study 8	Emotional Contagion	N/A	N/A	0 out of 10	Emotional Contagion
Study 9	Framing and Message Fit Theories	Elaboration Likelihood Model (ELM)	Context Fit, Source Credibility, Attribution Theory	3 out of 8	Elaboration Likelihood Model (ELM)
Study 10	-	N/A	N/A	0 out of 3	-

**Table A4:** Countries where experiments/analysis were conducted (n = 43).

Country of Experiment/Analysis	Number of Articles
USA	10
China	5
South Korea	4
Multi-national	4
Australia	2
Germany	2
India	2
Taiwan	2
Australia/Singapore	1
Austria	1
Canada	1
China/Hong Kong	1
China/USA	1
Colombia	1
Czech Republic	1
Iran/USA	1
Portugal	1
South Africa	1
Spain	1
Tunisia	1

**Table A5:** Industries of experiments/analysis (n = 27).

Industry	Number of Articles
Travel	6
Food	3
Ecommerce	2
Electronics	2
Health	2
Multiple	2
Banking	1
Beauty	1
Education	1
Facilities	1
Governmental	1
Luxury Fashion	1
Mineral Water	1
Retail	1
Social Media Commerce	1
Sports	1

**Table A6:** Independent variables(n = 35).

Independent Variable	Percentage of Studies	Number of Studies
Presence of Emoji	51.43%	18
Number of Emojis	8.57%	3
Emojis Ratio	5.71%	2
Not Applicable	5.71%	2
Brand Political Ideology	2.86%	1
Donation Size	2.86%	1
Emoji Asymmetry/Symmetry	2.86%	1
Emoji function/Type of Emojis	2.86%	1
Emoji Type	2.86%	1
Facebook Emoticon Reactions	2.86%	1
Message Source	2.86%	1
Number and Sentiment of Emojis	2.86%	1
Photo Editing Behavior	2.86%	1
Type of Content	2.86%	1

**Table A7:** Dependent variables (n = 35).

Dependent Variables	Number of Studies
User Engagement	8
Purchase Intention	4
Users/Customer Evaluations	3
Consumer Engagement	2
Brainwave Oscillations, Consumers’ Selection (ratings of hotels)	1
Communication of D&I, Engagement	1
Construal Level	1
Customer Satisfaction, Likeness, State Anxiety, Repurchase Intention, and Recommendation Intention/eWom	1
Customer Satisfaction/Repurchasing Behavior	1
Difference Between Implicit and Explicit Measures of Emotional Valence	1
Effectiveness of Pre-roll Advertising	1
Feelings of Social Presence, Evaluation of Customer Service Agent, Interaction Experience, and Task Impression	1
Instagram Likes	1
Message Evaluations, eWOM Volume	1
Not Applicable	1
Perceived Attractiveness of Influencer	1
Persuasion, Perceived Ambiguity	1
Positive Affect, Perceived Relationship Strength, and Future Purchase Intentions	1
Purchase Intention, Processing Fluency, Claim Believability, and Attitude Toward the Ad Attitude Toward the Brand	1
Purchase Intention, Review Trustworthiness	1
Social Media Engagement, Content-Based Trust, and Engagement-Based Trust	1
Valence of Message	1

**Table A8:** Moderators (n = 18 studies).

Moderating Variables	Frequency
Presence of Emoji	3
Type of Product (utilitarian/hedonic)	2
Commerce Feature	1
Emoji Skin Tone, Number of Emojis, and Nature of Communication (paid ads)	1
Emoji Type (normal vs. combination)	1
Follower Size	1
Function of Emoji: Replacement (substitution) or Reiteration, High/Low Content Richness	1
Nationality	1
Need for Cognition, Brand–Emoji Fit, and Advertising Objective (promoting products vs. social marketing)	1
Personalization	1
Profile Picture (presence/absence), Giver–Recipient Relationship	1
Relationship Norm	1
Relationship Type	1
Relevance of Emoji	1
Seller Quality	1

**Table A9:** Mediators (n = 15).

Mediating Variables	Frequency
Positive affect	1
Positive affect, CTR	1
Positive conscious affective reaction	1
Affective social presence	1
Emoji type (normal vs. combination)	1
Brand advocacy related to conservative beliefs	1
Emotional arousal, perceived ambiguity	1
Emotional response, cognitive response	1
Human expression resemblance	1
Level of narrative transportation + lower message comprehensibility + feelings of curiosity + higher sensations of humor + perceptions of message credibility and brand trustworthiness (childishness)	1
Perceived competence	1
Perceived intrusiveness	1
Perceived sincerity, willingness to forgive	1
Perceived usefulness, involvement, and perceived ease of use	1
Skepticism (openness)	1

**Table A10:** Sub-categories of experimental studies (n = 13).

Emoji Function	Number of Studies
Online Experiments	4
Lab Experiments	3
Multi-Study Experimental	2
Factorial Designs	1
Lab and Online Experiments	1
Lab and Field Experiments	1
Online and Field Experiments	1

**Table A11:** Sub-categories of mixed methods studies (n = 10).

Emoji Function	Number of Studies
Multi-Study Designs	5
Concurrent Mixed Methods	3
Sequential Mixed Methods	2

**Table A12:** Sub-categories of content analysis studies (n = 10).

Emoji Function	Number of Studies
Manual Content Analysis	8
Taxonomy Development	2

**Table A13:** Sample characteristics of experimental studies.

Study ID	Sample Size (N)	Age M ± SD (Range)	Gender Distribution	Population	Location	SLR No.
S1	122	37.48 ± NR	65.6% F, 34.4% M	General	USA	25
S2	250	NR (18–39 years, 62.8%)	46% M, 54% F	General	USA	16
S3a	194	23.4 ± 3.29	43% F, 57% M	Young adults	Taiwan	26
S3b	180	27.3 ± 2.19	45% F, 55% M	Young adults	Taiwan	26
S4	400	33.7 ± NR	50% F, 50% M	General	South Korea	27
S5	16	NR (24–32)	56.3% F, 43.7% M	General	Taiwan	33
S6	118	NR (62.7% <26)	59% F, 41% M	Young adults	South Korea	28
S7	280	39.96 ± NR	44.6% F, 55.4% M	General	USA	19
S8	217	24.38 ± 7.47 (18–60)	62.2% F, 30.4% M, 3.7% Trans/NB, 3.7% Undisclosed	General	International (30 countries)	20
S9	108	23.4 ± NR	56% F, 44% M	General	South Korea	43
S10	113	NR	57.5% F, 42.5% M	Undergraduates	USA	36
S11a	40	36.84 ± 13.51 (21–75)	50.6% F, 49.4% M	General	USA	42
S11b	90	38.00 ± 12.75 (20–75)	47.7% F, 52.3% M	General	USA	42
S12	1272	NR	NR	Generation Z	Czech Republic	22
S13	451	NR (18–35)	NR	Urban, educated	India	24
S14a	137	34 ± NR	44% F, 56% M	General	USA	39
S14b	169	23 ± NR	39% F, 61% M	General	USA	39
S15	169	21.6 ± NR	61% F, 39% M	General	China/USA	40
S16	30	NR (16–55)	53% F, 47% M	General	Spain	32
S17	1682	22.83 ± 3.31	55.1% F, 44.9% M	General	Germany	31
S18	104	33.74 ± NR	57.6% F, 42.4% M	General	USA	10
S19	132	22.50 ± 2.96	51% F, 49% M	General	Germany	11
S20	324	NR (95% aged 19–38)	66% F, 34% M	General	USA	14
S21a	93	NR	NR	NR	China	5
S21b	110	NR	NR	NR	China	5
S21c	102	NR	NR	NR	China	5
S22	1000	NR	NR	Generation Z	South Africa	8

**Table A14:** Characteristics: Key findings and future directions.

Variable/Category	Distribution of Studies	Key Findings	Future Directions
**Section 4.3.1 Independent Variables**			
**Emoji Presence/Absence**	Dominant independent variable (N = 18, >50% of studies)	Emoji presence **generally brings positive consumer responses**: Higher post engagement (Taba et al., 2023), affect transfer (Das et al., 2019; Smith & Rose, 2020; Neel et al., 2023), feelings of social presence (Park & Sundar, 2015), and customer selection (Hsu & Chen, 2020). **No consensus on purchase intentions**: Same product shows contradictory results (Das et al. 2019 vs. Lee et al., 2021). **Negative effects**: Hurt seriousness/authenticity in healthcare (Madadi et al., 2024).	Resolve contradictory findings about purchase intention for hedonic and utilitarian products; test self-reported vs. implicit measures alignment; and test if the observed results are valid for non-facial emojis.
**Quantitative** (number of emojis, facial emojis, emoji ratio)	2 studies each	“**More is not better**”: Higher numbers linked to higher review trustworthiness (Huang et al., 2021), optimal Twitter engagement at 0–3 emojis (Guzmán Ordóñez et al., 2024). More emojis hurt perceived competence (Orazi et al., 2023). No effect of emoji ratio (Yang & Shin, 2021).	Investigation of optimal emoji ratios; test across different content lengths and platforms.
**Categorical**	N = 3	Non-facial emojis increase eWOM volume (Orazi et al., 2023), asymmetrical facial emojis boost engagement (Hewage et al., 2021), negative sentiment emojis improve service recovery (Ma & Wang, 2021).	Explore systematic comparison across emoji categories; test how different types function in various contexts.
**Other Findings**		Most of the independent variables are about emojis. No attention at the antecedents of emoji usage by brands.	Explore other independent variables that affect emoji usage/selection by brands.
**Section 4.3.2 Dependent Variables**			
**User** **Engagement**	12 studies (34.29%)	**Positive effects**: Posts with emojis receive more likes (Taba et al., 2023); TikTok captions with emojis shared more (Einsle et al., 2024). Strategic dependence: Same emoji produces different results by topic (X. Wang et al., 2023a; J. Yu et al., 2024). Context-sensitive failures: **Less effective** in diversity/inclusion posts (Bombaij & Mokarram-Dorri, 2024); firm-generated content less effective than employee-generated due to expertise loss.	Test across different platforms and content types; develop emoji-topic matching guidelines; and investigate expertise perception mechanisms. Explore long-term effects.
**Purchase-Related Outcomes**	7 studies (20%)	Product type moderation: Stronger effects for hedonic vs. utilitarian products via trust and positive affect (Das et al., 2019; Mladenović et al., 2022; Duffett & Maraule, 2024). Relationship context matters: Facial emojis more effective in communal vs. exchange relationships (Huang et al., 2021). Contradictory evidence: Same product (camera) shows opposing effects in different contexts.	Expand beyond Generation Z samples; test utilitarian products.
**Specialized Measures**	5 studies (14.29%)	Sophisticated psychological mechanisms: Subliminal emoji cues (1ms) influence hotel selection (Hsu & Chen, 2020). More advanced variables: Brainwave oscillations, construal level, implicit vs. explicit valence measures reveal hidden responses.	With neuroimaging research, test attention and processing prior to engagement.
**Customer Evaluations**	4 studies (11.43%)	**Mostly positive effects on** review trustworthiness, hotel selection, employee communication, influencer attractiveness. **Intriguing finding**: Dark skin-tone emojis increase conservative brand preference through higher advocacy (Boman et al., 2023).	Explore cultural appropriateness.
**Section 4.3.3 Moderators**			
**Presence of Emojis**	N = 3 (16.7%)	Emojis as moderators: Moderate content-effectiveness relationships, reinforce positive thumbnail effects, influence source credibility perceptions.	Investigate complex boundary conditions.
**Congruence Factors**	N = 3	Brand-emoji fit, campaign objective-emoji fit, and content-emoji fit all moderate effectiveness (Rúa-Hidalgo et al., 2021; X. Wang et al., 2023b; C. H. Kim et al., 2024).	Explore semantic, emotional, and cultural congruence types; additional research for congruence of brand traits with types of emojis.
**Product Type**	N = 2	Product type consistently validated: Hedonic products show increased emoji effectiveness (Mladenović et al., 2022; Das et al., 2019).	Test with utilitarian products and with complex designs with different types of content. Use EASI model to explain affective and cognitive pathways in both types.
**Relationship Variables**	N = 3	Communal vs. exchange relationships: Higher emoji effects in communal relationships for affect, satisfaction, and repurchasing (Smith & Rose, 2020; Ma & Wang, 2021). Emojis provide fewer benefits to premium sellers (Orazi et al., 2023).	Test relationship variables; explore emoji effectiveness in luxury brands.
**Cultural**	N = 1	Cultural moderation unclear: No differences found between 3 Western countries (Neel et al., 2023).	Test across diverse cultural contexts.
**Section 4.3.4 Mediators**			
**Affective**	N = 7 mediators	Emotional pathways dominate: Positive affect, emotional arousal, anthropomorphic nature, pleasure, all facilitate emoji effectiveness, and primarily for facial emojis (Smith & Rose, 2020; Hewage et al., 2021; X. Wang et al., 2023a).	Test negative affect in appropriate contexts; compare affective vs. cognitive pathways; and for valence and arousal, test different times of day.
**Cognitive**	N = 9 mediators	Mostly suppressing effects: Processing fluency, ambiguity, comprehensibility, skepticism, perceived competence, and childishness (C. H. Kim et al., 2024; Orazi et al., 2023). Facilitating effects: Perceived usefulness, perceived ease of use and involvement. May suit semantic emojis better.	Test more cognitive variables as facilitating mediators; replicate for perceived usefulness, ease of use, and involvement; investigate semantic processing mechanisms; and examine attention, comprehension, and memory variables.
**Social**	N = 3 mediators	Relationship mechanisms: Affective social presence, perceived sincerity, and willingness to forgive mediated effects. Service recovery: Negative emojis increase satisfaction through sincerity/forgiveness (Ge & Gretzel, 2018).	Test across different relationship contexts; explore service failure emoji strategies; and investigate social presence enhancement.
**Other Mediators**	N = 4 mediators	Click-through rate, emoji type (normal vs. combination), brand advocacy, and level of narrative transportation. Very scattered across many variables.	Consistently replicate.
**No Mediation**	57.1% of studies	Critical methodological gap: Over half examine no mediating variables, severely limiting mechanism understanding.	Develop comprehensive mediation frameworks; test multiple mediator models.
**Other characteristics**			
**4.3.5 Emoji Frequency and Combinations**	13 studies reporting: 53.85% single emoji, 23.08% multiple	**Contradictory quantity effects**: More emojis hurt perceived competence but improve review trustworthiness (Orazi et al., 2023; Huang et al., 2021). User interest in combinations: Rare combinations found “very interesting” (Guo & Wang, 2024)	Test combinations and frequency systematically; resolve discrepancies between studies; and frequency specification in all studies.
**4.3.6 Emoji Placement**	23 studies reporting: 9 studies (39.13% of reporting studies) at sentence end; 0 at beginning	**Sentence-final preference**: Reflects linguistic research on semantic benefits (Grosz et al., 2023). Supplement vs. replacement: **Generally better processing when supplementing vs. replacing** words, but context exceptions exist (C. H. Kim et al., 2024).	The beginning of sentence to be tested; investigate placement effects systematically.
**4.3.7 Emoji Sentiment**	11 studies reporting: 63.6% positive only, 0% negative only (of 11 reporting studies)	**Clear research bias**: Overwhelming focus on positive emojis. **Negative sentiment benefits**: Service recovery context shows negative emojis increase satisfaction/repurchasing through perceived sincerity (Ma & Wang, 2021).	Overcome positivity bias; test negative sentiment in appropriate contexts; explore urgency/scarcity messaging; and examine brand vs. user usage patterns
**4.3.8a Emoji Type**	19 studies reporting: 61.54% facial only	“Anthropomorphic bias”: Focus on human-like emojis due to face detection wiring (Weiß et al., 2020), different text processing vs. non-facial (Cao et al., 2024). Research-practice gap: Popular non-facial emojis (hearts, symbols) underrepresented vs. real usage patterns.	Test non-facial emojis; systematic type comparisons; align research with actual usage patterns; test EASI theory validity for non-facial emojis; expand beyond facial emoji focus;
**4.3.8b Emoji Function**	20 studies reporting: 35% emotional only, 35% combined with other functions	Function research gaps: No studies explore only semantic or navigational/emphasis/decoration functions. Research vs. practice mismatch.	test semantic aspects; investigate navigational/emphasis and decoration functions; align research with brand emoji preferences; and test functional effectiveness across contexts.
